# Substitution of copper atoms into defect-rich molybdenum sulfides and their electrocatalytic activity†

**DOI:** 10.1039/d0na01064b

**Published:** 2021-01-26

**Authors:** Zixing Wang, Harikishan Kannan, Tonghui Su, Jayashree Swaminathan, Sharmila N. Shirodkar, Francisco C. Robles Hernandez, Hector Calderon Benavides, Robert Vajtai, Boris I. Yakobson, Ashokkumar Meiyazhagan, Pulickel M. Ajayan

**Affiliations:** Department of Materials Science and NanoEngineering, Rice University Houston TX 77005 USA ma37@rice.edu ajayan@rice.edu; Department of Mechanical Engineering Technology, University of Houston Houston Texas 77204-4020 USA; Departamento de Física, ESFM-IPN, Ed. 9, Instituto Politécnico Nacional UPALM Mexico D.F. 07738 Mexico; Interdisciplinary Excellence Centre, Department of Applied and Environmental Chemistry, University of Szeged Rerrich Béla Tér 1 Szeged H-6720 Hungary; School of Materials Science and Engineering, Beihang University Beijing 100091 P.R. China

## Abstract

Studies on intercalation or substitution of atoms into layered two-dimensional (2D) materials are rapidly expanding and gaining significant consideration due to their importance in electronics, catalysts, batteries, sensors, *etc.* In this manuscript, we report a straightforward method to create sulphur (S) deficient molybdenum (Mo) sulfide (MoS_2−*x*_) structures and substitute them with zerovalent copper (Cu) atoms using a colloidal synthesis method. The synthesized materials were studied using several techniques to understand the proportion and position of copper atoms and the effect of copper functionalization. Specifically, the impact of change in the ratio of Cu : S and the hydrogen evolution reaction (HER) activity of the derived materials were evaluated. This technique paves the way for the synthesis of various functionalized 2D materials with a significant impact on their physical and chemical behavior making them potential candidates for catalysis and several other applications such as energy storage and the development of numerous functional devices.

## Abstract 2

Colloidal technique was used to synthesize copper atom substituted molybdenum sulfides and used as a catalyst. The derived materials were characterized using several analytical methods and revalidated using DFT calculations.

## Graphical abstract

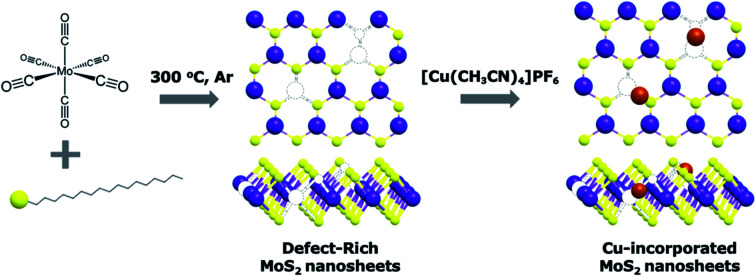

## Introduction

Two-dimensional transition metal dichalcogenides (2D TMDCs) have gained significant interest due to their versatile and unique electrical, optical, chemical, and mechanical properties, with practical applications in electrocatalysts, photocatalysts, energy storage, and optoelectronics.^1–4^ In general, TMDCs are layered materials represented by MX_2_, where a layer of transition metal atoms (M) is sandwiched between two layers of chalcogen atoms (X). To date, 40 different types of layered TMDCs exist, with sulphur, selenium, and tellurium forming layered compounds with nine metals from group IV, V, VI, VII, and IX (Ti, Zr, Hf, V, Nb, Ta, Mo, W, Tc, Re, Pd, and Pt) and with Co, Rh, Ir, and Ni partially.^5^ In particular, 2D MoS_2_ has shown great potential to function as an economic HER catalyst due to its relatively moderate Gibbs free energy (*i.e.*, atomic hydrogen bond formation is neither too strong nor too weak).^6–8^ However, these 2D materials have a significant portion of the inert basal plane, reducing the number of active sites and electrical conductivity, thus limiting their application in practical hydrogen production.^9^

Nevertheless, several activities are being carried out to overcome these obstacles by modulating their composition, crystal structure/phase transformation, and morphology.^10^ Even though these reformations aim to enhance the active sites, they involve complex techniques, high cost, and undesirable side reactions, reducing the material's stability. To overcome these consequences, chemical doping of TMDCs is considered an attractive and promising pathway to activate the basal planes.^11^ During this process, transition metal atoms were intermingled with TMDCs using different techniques to increase active site density on the basal plane.^12^ However, most commonly, the incorporation of transition metal atoms into the TMDC layers was carried out through substitution, adsorption, and inter-layer intercalation.^13–16^ To validate, previous studies involving first-principles computations using density functional theory (DFT) have shown the favorable substitution of MoS_2_ by group III–VI transition metals.^17^ Doping and alloying MoS_2_ with transition metals from these groups through substitution have been widely studied^18,19^ and carried out commonly through chemical vapor deposition (CVD) and sputtering techniques.^12,20^ However, group VII to IB transition metals (such as copper, gold, *etc.*) have high formation energies *i.e.* unfavorable reaction conditions since M-rich MoS_2_ would prefer S substitution, while an X-rich structure would prefer M substitution.^17^

On the other hand, atomic copper (Cu) has shown various promising applications, including notable catalytic activities.^21,22^ Anchoring Cu atoms with MoS_2_ through substitution of S atoms might provide a stable Cu doped MoS_2_ structure. For example, S vacancy substitution with group IB metal was predicted to increase the number of active sites in the MoS_2_ monolayer, making it a suitable candidate for nucleophilic and electrophilic attacks.^23^ Similarly, the energy for Cu substitution at both X or M-sites is ∼2 eV, under M-rich and X-rich conditions, respectively, hence suggesting relative ease of substitutional doping.^17^ The theoretical calculations of the adsorption energy of Cu atoms on the MoS_2_ surface was estimated to be 1.3 eV, signifying the possibility of Cu atom adsorption.^24^ Hence, several attempts have been carried out to dope or intercalate MoS_2_ with group VII to IB transition metals using the hydrothermal technique.^25–28^ More importantly, the Cu-doped MoS_2_ on CdS nanorods displayed a 52-fold enhancement in photocatalytic hydrogen production.^29^ Similarly, the electrodeposition of Cu onto MoS_2_ has been reported to derive Cu doped MoS_2_ thin films, but the derived structure is unclear.^30^ However, changing the chemical potentials of the elements alters the formation energies, and doping MoS_2_ with transition metals through substitution with high formation energy is considered feasible.

A report on Cu-doped MoS_2_ using the hydrothermal synthesis technique found that substitution of Mo with Cu atoms influences magnetism strongly and results in enhanced hydrogen evolution reaction.^31^ However, in general, the hydrothermal method possesses certain limitations such as repeatability, consistency, and control over crystal growth. Another issue associated with heteroatom doping is the instability of the coordinatively unsaturated edge atoms. As defective MoS_2_ is intrinsically metastable due to its inherent high surface energy, it suffers from a high sulfur leaching rate. Herein, we resolve these problems concurrently through a simple wet-chemical method to synthesize Cu-substituted MoS_2_ nanostructures. In brief, a Cu metal–ligand compound (tetrakis(acetonitrile)copper(i) hexafluorophosphate) was used as a precursor for the synthesis of Cu atom substituted MoS_2_ nanostructures. It is found that tetrakis(acetonitrile)copper(i) hexafluorophosphate leads to Cu atom doped MoS_2_ nanostructures with Cu atoms occupying S positions within the MoS_2_ lattice. A careful evaluation of the derived samples was carried out using different analytical techniques to understand the nature of copper in the synthesized MoS_2−*x*_ framework. Our observation suggests that this method demonstrates a highly controllable Cu concentration with the advantage of easy scalability and repeatability for application in catalysis and other areas such as electronics and energy storage.

## Results and discussion

**Fig. 1:**
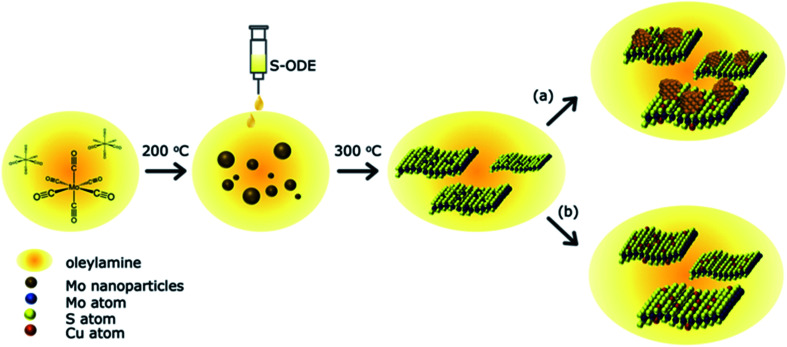
Overview of the reaction of Cu doped MoS_2−*x*_. Mo(CO)_6_ was dissolved in oleylamine and reduced to Mo(0) at 200 °C. The S-deficient MoS_2−*x*_ was synthesized by injecting a 1.5 times the molar amount of S-ODE into the Mo solution and reacted at 300 °C. The incorporation of tetrakis(acetonitrile)copper(i) hexafluorophosphate yields atomically substituted Cu–MoS_2−*x*_ as shown in (b) as well as copper aggregates as displayed in (a) under different reaction conditions.

A schematic of the reaction between the Cu, Mo, and S precursors is shown in Fig. 1. The detailed experimental procedure is discussed in the materials and methods section. In brief, Oleylamine (OLA) acts as both a high-temperature solvent and a reducing agent. 1-Octadecene (ODE) was used as a dopant to atomically disperse S atoms into the solution and hence lowers the energy of activation for the spontaneous formation of MoS_2−*x*_ nanostructures. Previous studies have shown control over the morphology of TMDCs with the addition of different surfactants.^32^ We purposefully reduced sulfur's molar ratio in MoS_2_ to create S vacancies within the nanostructures, followed by Cu precursor addition. After completion of the reaction, the end products were washed with an excess of organic solvents and annealed to remove residual surfactants. The current process has a ∼10% yield, which is easily scalable compared to chemical vapor deposition methods. The derived products were investigated to understand the Cu atom bonding state and location in the synthesized MoS_2−*x*_ nanoparticles. The first section discusses the atomic substitution of Cu atoms within the MoS_2_ lattice at a low Cu concentration. The second part investigates the influence of higher Cu concentrations and various Cu decomposition temperatures and their related products.

### Section 1: product characterization

**Fig. 2:**
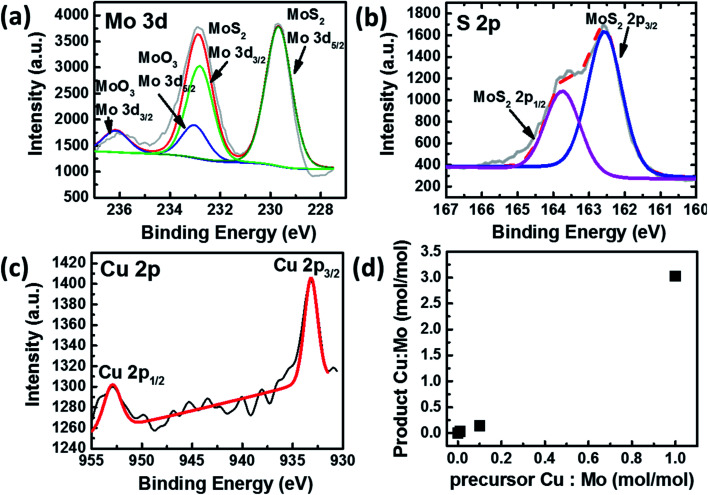
(a) Mo 3d XPS spectra and fittings of MoS_1.99_Cu_0.01_. (b) S 2p XPS spectra and fitting of MoS_1.99_Cu_0.01_ (c) Cu 2p XPS spectra and fittings of MoS_1.99_Cu_0.01_. (d) Correlation between the Cu : Mo molar ratio in the precursor and the product.

**Fig. 3:**
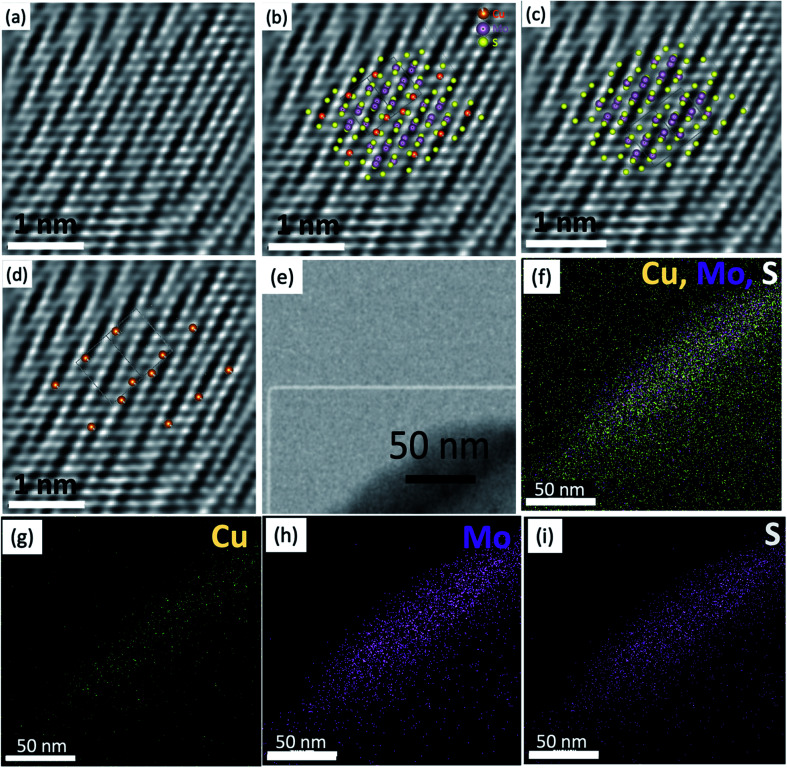
TEM analysis of the MoS_1.99_–Cu_0.01_ and respective EDS overlapped map of individual elements: Cu, Mo, and S (a) shows the STEM image of the synthesized material. (b) The atomic resolution image showing a crystalline domain-oriented in the zone axis [101]. (b–d) show the IFFTs of (b) showing the match among the experimental atomic resolution image and the simulated MoS_1.99_Cu_0.01_ (c) MoS_1.99_Cu_0.01_ without the Cu atoms and (d) MoS_1.99_Cu_0.01_ showing the Cu atoms in the lattice. (e) Low magnification of the analyzed region, (f) EDS mapping displaying Cu, Mo, and S and (g–i) exhibit Cu, Mo and S separately for a better resolution. (Scale bar = 50 nm).

The elemental composition and bonding states of Cu doped MoS_2_ were studied using the XPS technique, which shows Mo, S, and Cu, as shown in (Fig. 2a–c and S1–S5†). The Mo and S spectra corresponding to MoS_1.99_Cu_0.01_ (Fig. S2†) are similar to those of pristine MoS_2−*x*_ (Fig. S1†). The S 2p_3/2_ peak (Fig. 2b) observed at 162.6 eV coincides with the previous report.^33^ We observed two prominent 3d peaks for Mo, one with 3d_5/2_ observed at 229.7 eV, which corresponds to Mo from MoS_2−*x*_, and is found to be around 80 at% of Mo concentration, and the other 3d_5/2_ peak is located at 233.0 eV (Fig. 2a). The molar ratio of Mo^4+^ : S^2−^ : Cu(0) or (I) is 0.29 : 0.69 : 0.01 for Cu doped MoS_2_ nanoparticles. We further carried out EDS mapping to confirm the incorporation of Cu into the MoS_2−*x*_ matrix. The EDS mapping elucidates high homogeneity with the precise distribution of Mo, S, and Cu atoms, all observed with good dispersion (Fig. 3).

It is important to note that EDS mapping was carried using a nickel (Ni) grid; therefore, the Cu detected by EDS mapping corresponds entirely to the atomic Cu present in the sample. The atomic resolution images (Fig. 3a–d) show the presence of nanostructured crystals with sizes between 2 and 10 nm and displays an apparent similarity among the Vesta® simulated structures and the experimental work. The observed image presents a series of continuous planes with gaps in-between (Fig. S6†); these gaps are directly associated with the presence of Cu atoms that is observed when comparing the simulated images and the experimental work. The reason for appearance as gaps instead of the actual atoms is the Z contrast typical of the EM technique. The brightness increases exponentially, usually quadratic, with the atomic weight.^34–36^ We investigated several crystals; however, the copper's location is easier to identify or it is isolated along the zone axis [101]. This orientation shows that the (101) plane is the only axis that indicates the presence of isolated Cu atoms, or else they are clustered with Mo and S.

The Vesta projections show a close approximation of the experimental observations. The differences between the experimental and the projections are less than 5%, as measured based on the Vesta simulated XRD and their respective CIF files. The copper is located within the regions with a crack like appearance, as seen in Fig. S6.† The Cu atoms generate these cracks, and the densely packed planes are primarily Mo–S clusters. Based on the respective atomic weight, the clusters weigh between 220 g mol^−1^ (Mo–S–Mo) and 158 g mol^−1^ (S–Mo–S) (Fig. 3b and c), whereas an independent copper atom weighs only 63.5 g mol^−1^. Therefore, the Mo–S–Mo or S–Mo–S clusters are significantly more massive; and hence brighter than the pristine Cu atoms (Fig. 3d). To further confirm the Cu atoms' precise location, we measured the distances between the atoms in different projections, and the results were compared to those in the experimental observations. Additional studies are displayed in the ESI (Fig. S7–S9†). For instance, the distance between the Cu atoms is approximately 0.994 nm.

Furthermore, in the [101] direction, the Cu atoms are found every 0.99 nm. Simultaneously, the Mo–S–Mo or S–Mo–S clusters are present every 0.27 and 0.39 nm, which is comparable to the experimental observations (Fig. S7†). This allows us to conclude that the dark bands or “empty planes” allocate the Cu atoms. In conclusion, Cu atoms substitute a Mo–S pair in the MoS_2_ lattice. Other potential effects contributing to the lower contrast to observe the Cu atoms could be associated with defocus and wave extinction.^25^ The surface morphology of the synthesized material was further observed using SEM, which shows a continuous sheet-like structure composed of nanometer-sized sheets (Fig. S10a†). The low-resolution TEM image of MoS_1.99_Cu_0.01_ displays a layered crystalline structure at the cluster's edges (Fig. S10b†). The Cu concentration in the samples prepared at different temperatures ranging from 25 to 300 °C increases with temperature, signifying temperature as one of the crucial factors in providing activation energy for the formation of Cu doped MoS_2_ (Fig. S11†).

**Fig. 4:**
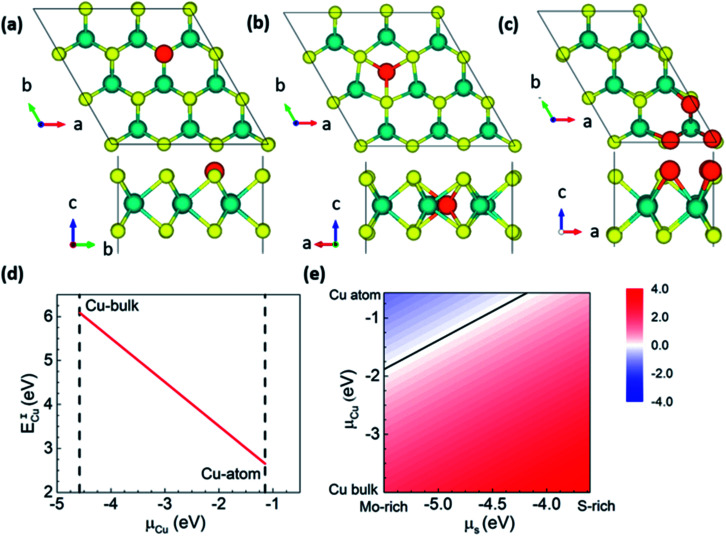
Top and side view of the atomic structures of (a) Cu intercalation and (b) S-substitution in MoS_2−*x*_Cu_*x*_, *x* = 1/9. (c) MoS_2−*x*_Cu_*x*_ at *x* = 1/2 top and side views of the most stable configuration for S-substitution by Cu. Color scheme, Cu: orange, S: yellow and Mo: blue. Variation in the formation energy of (d) Cu intercalation *E*^I^_Cu_ and (e) S-substituted *E*^S^_Cu_ for variation in chemical potentials, *μ*_S_ and *μ*_Cu_ for *x* = 1/9. The line in (e) represents *E*^S^_Cu_ = 0 eV.

To confirm the feasibility of S-substituted Cu doping of MoS_2_, we carried out first-principles calculations to estimate the formation energies. We considered both interstitial and substitutional doping to understand Cu doping's position and energetic stability in MoS_2_ (Fig. 4). Since the experimental conditions are Mo-rich, the Mo-substitution is highly energetically unstable compared with S-substitution,^17^ and hence we only consider the latter case. S-substitution doping (*E*^S^_Cu_) formation energy and interstitial doping (*E*^I^_Cu_) were calculated using the following expressions.

1 *E*^S^_Cu_ = *E*(*n*MoS_2−*x*_Cu_*x*_) − [*nE*(MoS_2_) − *nxμ*_S_ + *nxμ*_Cu_]

**Fig. 5:**
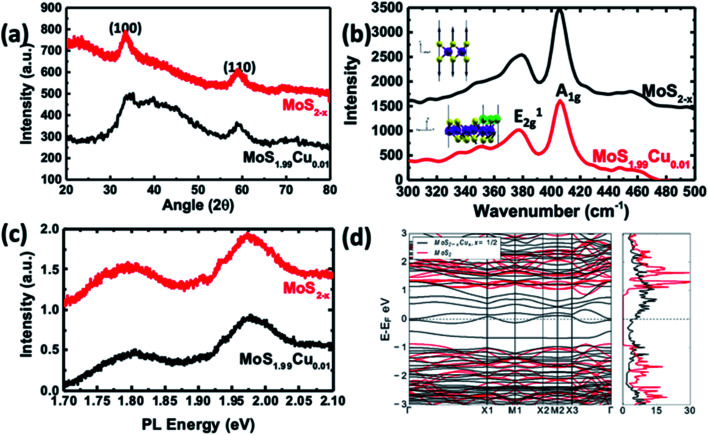
Characterization of MoS_1.99_Cu_0.01_ compared to MoS_2−*x*_. (a) XRD pattern and (b) Raman spectra. (Top inset) Phonon mode corresponding to the Raman peak in undoped MoS_2_. (Bottom inset) The Phonon mode of a shifted peak in the structure is due to Cu substitution. We observed the out-of-phase displacement of S atoms in the ‘*z*’ direction for Cu–MoS_2_ and it has the largest overlap with the pristine mode shown in pristine MoS_2_. (c) PL spectra of the synthesized materials. (d) Calculated electronic band structure and density of states of the most stable configuration (black lines) shown in Fig. 2d compared with those of undoped MoS_2−*x*_ (red lines).

2 *E*^I^_Cu_ = *E*(*n*MoS_2_Cu_*x*_) − [*nE*(MoS_2_) + *nxμ*_Cu_]

where *E*(*n*MoS_2−*x*_Cu_*x*_) is the energy of the doped MoS_2−*x*_Cu_*x*_ supercell with *n* formula units, and *E*(MoS_2_) is the energy of a primitive undoped MoS_2_ cell. *μ*_S_ and, *μ*_Cu_ are the chemical potentials of S and Cu, respectively. The chemical potential of S under Mo-rich conditions is calculated as, *μ*_S_ = *E*(MoS_2_) − *E*(Mo in bulk), whereas under S-rich conditions, it is obtained from the diatomic S_2_ molecule. We constructed a 3 × 3 × 1 supercell with 1 Cu atom doping for low doping concentrations, which corresponds to MoS_2−*x*_Cu_*x*_, *x* = 1/9 (MoS_1.88_Cu_0.11_, see Fig. 5). *E*^S^_Cu_ varies between 2.12 eV and 4.02 eV between Mo-rich to S-rich conditions and is in good agreement with previous reports^17^ (Fig. 5c). However *E*^I^_Cu_ ∼ 6 eV (*μ*_Cu_: bulk) is considerably higher than *E*^S^_Cu_, and hence is an indicator of the difficulty of doping Cu interstitially in MoS_2_. We present the variation in formation energies w.r.t. to the chemical potentials of S and Cu in Fig. 5d and e, where *μ*_S_ is varied between Mo-rich and S-rich conditions and, *μ*_Cu_ between its bulk and atomic form. We find that Cu in the interstitial site is always energetically higher in energy w.r.t. S-substitution. Besides, under Cu-atomic and Mo-rich conditions, we find that *E*^S^_Cu_ < 0, *i.e.*, Cu spontaneously occupies the S site, creating many doped states. The area above the black line in Fig. 5e is the S-substituted phase, and the area below represents undoped pristine MoS_2_. It is important to note that under these S and Cu rich/poor conditions the Cu interstitial phase get stabilized, and MoS_2_ remains undoped.

We also focused on higher concentrations of Cu doping for the S-substitutional case since the experiment corresponds to Mo-rich conditions with highly reduced stoichiometry (*i.e.*) *x* = 0.5. To find out the most probable and energetically stable distribution of Cu atoms in the S-lattice, we simulate all possible symmetry inequivalent configurations of Cu substitutions using the site occupancy disorder code.^37^ For the 2 × 3 × 1 supercell *x* = 0.5 corresponds to 3 Cu dopants with 19 symmetry inequivalent configurations. An increase in the supercell size, for instance, to 3 × 3 × 1 corresponds to approximately 500 symmetry inequivalent configurations, which are currently out of the scope of this work. The 2 × 3 × 1 supercell captures most of the essential Cu co-ordinations that can be expected with larger cells. The most stable configuration corresponds to Cu clustering on the same plane of S atoms (see Fig. 4c), which corresponds to formation energies of −1.9 (*μ*_Cu_: atomic Cu) and 1.6 (*μ*_Cu_: bulk Cu) eV per Cu atom under Mo-rich conditions. It is stable by 87 meV per Cu compared to the second-most stable configuration (>RT, the Boltzmann energy at room temperature), *i.e.*, Cu substituting S sites will prefer clustering, which is in agreement with our experimental results. Our Bader charge analysis^38^ shows that Cu is in the 0+ state in the MoS_2_ lattice (see Fig. 4), and the metallicity of Cu substitution is depicted in the band structure. Hence, confirming that Cu substitution at S sites is preferred under the experimental conditions, and the samples that we synthesized are indeed Cu substituted at S sites in MoS_2_.

### Section 2: reaction conditions

All characterization and calculations in the previous section were performed on MoS_1.99_Cu_0.01_ with tetrakis(acetonitrile)copper(i) hexafluorophosphate decomposed at 100 °C. Furthermore, reaction conditions, including precursor ratios and the reaction temperature were varied, and three different Cu to Mo ratios were derived 0.01 : 1, 0.1 : 1, and 1 : 1, and the final products are denoted as MoS_1.99_Cu_0.01_, MoS_1.9_Cu_0.1_, and MoS_1_Cu_1_. Tetrakis(acetonitrile)copper(i) hexafluorophosphate was decomposed at 4 different temperatures of 25, 100, 200, and 300 °C.

The XPS spectra distribution was observed with various Cu doping concentrations MoS_1.9_Cu_0.1_, and MoS_1_Cu_1_. The peaks observed at the same location indicates a consistent elemental bonding, regardless of the Cu concentration (Fig. S3–S5†). The concentration of Cu in the Cu doped MoS_2_ increases with an increase in the amount of tetrakis(acetonitrile)copper(i) hexafluorophosphate in the precursor (Table 1 and Fig. 2d).

**Table 1:** Mo^4+^ : S^2−^ : Cu(0) or (I) molar ratio in the MoSCu compound product prepared at different precursor ratios and temperature

Sample	*T*_Cu decomposition_ (°C)	Mo^4+^ : S^2−^ : Cu
Colloidal MoS_2_ nanostructures	NA	0.39 : 0.61 : 0
MoS_1.99_Cu_0.01_	100	0.29 : 0.69 : 0.01
MoS_1.9_Cu_0.1_	100	0.34 : 0.61 : 0.05
MoS_1_Cu_1_	100	0.15 : 0.41 : 0.44
MoS_1_Cu_1_	200	0.18 : 0.44 : 0.38

We observed a three-time increase in the product ratio of Cu : Mo after the completion of the reaction. For example, in the MoS_1.99_Cu_0.01_ sample prepared at 100 °C, the Mo to Cu molar ratio was 1 : 0.01 in the precursor. However, we observed a Mo to Cu molar ratio of 1 : 0.03 in the final product. This indicates a consistent doping efficiency of Cu with precursor concentration and robust controllability in the synthesis. As seen through XPS, we observed two kinds of oxide (MoO_3_ and CuO) in the final product. Their molar ratio compared to that of Cu doped MoS_2_ is shown in Table S1.† The MoO_3_ formation is independent of the Cu doping process. It is a byproduct of MoS_1.5_ synthesis since excessive Mo precursor was used during the synthesis. Similarly, CuO was only observed in MoS_1_Cu_1_ when a large amount of Cu is present, and with a higher Cu decomposition, more CuO formation was observed. However, the sample's CuO amount is relatively small, with a 0.07 : 1 ratio with Cu doped MoS_2_.

Fig. 5a illustrates the XRD crystallographic structure of MoS_2−*x*_ nanoparticles and MoS_1.99_Cu_0.01_. The MoS_2−*x*_ nanoparticles show two distinct peaks at 33.3° and 59.2°, which correspond to (100) and (110) planes.^39^ Interestingly, we did not observe the (002) peak in the synthesized MoS_2−*x*_, due to the formation of well-separated individualized nanosheets. On the other hand, the XRD spectra of MoS_1.99_Cu_0.01_ did not display additional noticeable peaks regardless of the reaction temperature (Fig. S12†). The absence of Cu metal peaks in the XRD confirms the nonexistence of Cu crystals in the synthesized MoS_1.99_Cu_0.01_, signifying the existence of Cu existence as distinct atoms. However, when the ratio of Cu was increased (*i.e.*, Cu : Mo = 1 : 1 mol mol^−1^), we observed a sharp XRD peak of Cu atoms in all the synthesized samples (Fig. S12e–g†). Notably, the sample synthesized at 100 and 200 °C displays prominent Cu peaks (see Fig. S12f and g†), indicating 100 °C as sufficient temperature for the decomposition of tetrakis(acetonitrile)copper(i) hexafluorophosphate. However, at an elevated temperature ∼300 °C, the peaks corresponding to MoS_2_ and Cu start to disappear, whereas the peaks analogous to CuS become dominant (Fig. S13a and b†). This suggests the instability of the synthesized material at 300 °C yielding CuS, instead of Cu incorporated MoS_2_.

Raman studies were carried out on the samples synthesized with different proportions of Cu incorporation such as (MoS_1.99_Cu_0.01_, MoS_1.9_Cu_0.1_, and MoS_1_Cu_1_), and at various temperatures (25, 100, and 200 °C). Fig. 5b shows the Raman spectra of MoS_2−*x*_, and Cu doped MoS_1.99_Cu_0.01_ synthesized at 100 °C. A dominant Raman peak was observed around 405.8 cm^−1^ for MoS_2−*x*_, which correlates with the A_1g_ vibration mode and a weak signal at 379.0 cm^−1^ corresponds to the E_2g_ vibration mode (Fig. 5b). Similarly, the MoS_1.99_Cu_0.01_ sample displays a peak at almost the same position of A_1g_ and E_2g_ peaks, indicating an undisrupted structure with a low percentage of Cu doping at 100 °C (Fig. 5b). However, we observed a slight shift in the A_1g_ peaks to a lower wavenumber for the Cu doped MoS_2−*x*_ with an increase in the concentration of Cu and reaction temperature, which eventually leads to a rise in buckling of the sheets (Fig. S14†) (Table 2).^40^

**Table 2:** Raman A_1g_ peak positions of Cu doped MoS_2_ prepared at various Cu concentrations and decomposition temperatures

*T*_Cu decomposition_ (°C)	A_1g_ peak position (cm^−1^)	
	MoS_1.99_Cu_0.01_	MoS_1_Cu_1_
25	405.82	404.86
100	405.80	403.90
200	405.74	402.40

The A_1g_ peak was relatively unshifted for the sample synthesized at lower Cu concentration. For example, the MoS_1.99_Cu_0.01_ sample synthesized at 100 and 200 °C (Fig. S14b and c†) shows the shift in the A_1g_ peak of 0.02 and 0.08 cm^−1^. At an identical reaction temperature (∼100 °C), the A_1g_ peak for MoS_1.9_Cu_0.1_ is observed at 404.0 cm^−1^ (Fig. S15c†), and for MoS_1_Cu_1_ is seen at 403.9 cm^−1^ (Fig. S16c†). The shift in the A_1g_ peak with the reaction temperature is most prominent with MoS_1_Cu_1_ (see Fig. S16d†). When copper was introduced at 25 °C, the A_1g_ peak is observed at 404.9 cm^−1^, which shifts left by 0.9 cm^−1^ compared to the unreacted MoS_2−*x*_. Moreover, increasing the temperature shifts the A_1g_ peaks of MoS_1_Cu_1_ by 1.9 cm^−1^ at 100 °C and 3.4 cm^−1^ at 200 °C. The positive correlation between the shift of the A_1g_ peak with the Cu amount in the sample confirms the shift from Cu. This shift can be attributed to increased Cu substitution, causing a negative strain in the MoS_2_ lattice as predicted.^17^ These observed Raman signatures were further evaluated using theoretical calculations. Additionally, SEM EDAX characterization was carried out to evaluate the presence of copper and the results are shown in Fig. S17–S19.†

To verify the softening of the A1 phonon mode, which corresponds to out-of-plane S displacements, we carried out DFT calculations to estimate the vibrational phonon frequencies at the Brillouin zone center for the most stable S-substituted Cu doped structure. We observed shifts from 410 cm^−1^ to a lower wavenumber at 347 cm^−1^ (Fig. 5d). Though the shift's magnitude did not quantitatively agree with experiments, our results precisely projected the direction of the shift and confirm its origin to Cu substitution in S sites. The slight disagreement between the DFT and calculated values might be because the calculated Raman signals are for a single unit cell.

The photoluminescence (PL) spectra of pristine MoS_2−*x*_ and MoS_1.99_Cu_0.01_ are shown in Fig. 5c and S20.† Two prominent signals were observed ∼1.97, and 1.80 eV, which correspond to A1 and B1 direct excitonic transition between the minimum of the conduction band and the splitting valence band spin–orbital coupling and the *K* point are observed in both samples.^41^ The existence of this direct exciton transition indicates a monolayer structure of MoS_2−*x*_ nanosheets. The substitution of Cu with a 0.01 molar ratio did not change the bandgap of the derived product. Furthermore, as seen through PL spectra, incorporating Cu into the MoS_2_ lattice did not alter the size of the bandgap due to a change in the Cu concentration and reaction temperature (Fig. S21–S22†).

**Fig. 6:**
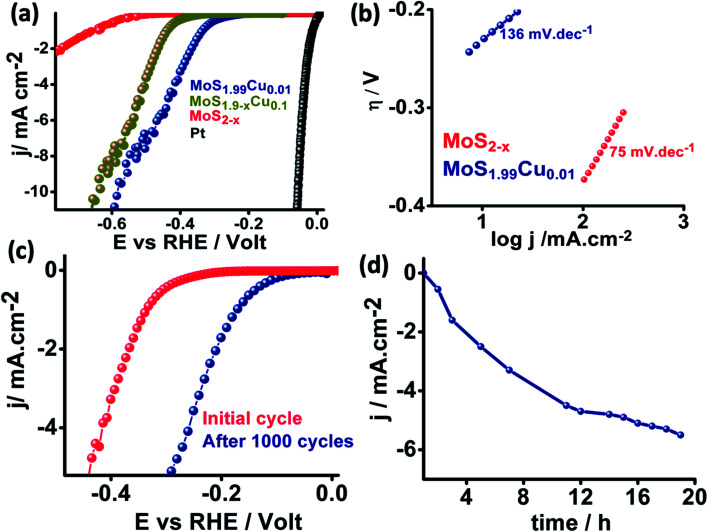
(a) Electrochemical linear sweep voltammogram of MoS_2−*x*_, MoS_1.99_Cu_0.01_ and MoS_1.9_Cu_0.1_ toward the HER in 0.5 M H_2_SO_4_ (b) Tafel plots of the corresponding catalysts (c) cyclic durability study and (d) chronoamperometry of MoS_1.99_Cu_0.01_ towards the HER.

We further examined the electrocatalytic HER behavior of the synthesized MoS_2−*x*_, MoS_1.99_Cu_0.01_, and MoS_1.99_Cu_0.1_ with benchmark platinum (Pt) in a 0.5 M H_2_SO_4_ medium, and the corresponding linear sweep voltammograms are shown in Fig. 6a. We observed that MoS_2−*x*_ displays a high onset potential of −0.58 V while the MoS_1.99_Cu_0.01_ exhibited enhanced HER performance and displays a lower onset potential of −0.26 V and an overpotential of −0.57 V at 10 mA cm^−2^, relatively close to those of the benchmark Pt (∼0 mV onset potential) and MoS_1.9−*x*_Cu_0.1_ shows an onset potential of 0.41 V and overpotential of 0.65 V at 10 mA cm^−2^. This observed performance is evidently superior and comparable to the performance of reported heteroatom-doped MoS_2_-based catalysts (Table S2†). The Nyquist plot derived from electrochemical impedance spectroscopy (Fig. S23†) shows enhancement in the performance of MoS_1.99_Cu_0.01_, which is due to a large reduction in charge transfer resistance from 480 000 Ω for MoS_2−*x*_ to 620 Ω for MoS_1.99_Cu_0.01_. Meanwhile, the Tafel plot (Fig. 6b) suggests that 0.01% of Cu doping is sufficient to enhance the electrochemical kinetics and reduce the Tafel slope from 136 to 75 mV dec^−1^. This demonstrates the better HER performance of MoS_1.99_Cu_0.01_, which might preferably be due to enriched active sites and hence transition of the rate-determining step towards an electrochemical desorption oriented Volmer–Heyrovsky mechanism.

We carried out potential cyclic sweeping and chronoamperometry to understand the electrochemical operation's extended stability performance, and the corresponding results are shown in Fig. 6c and d. The potential cyclic sweeping was performed in a range between 0 and −0.45 V *vs.* RHE (Fig. 6c) with a scan rate of 10 mV s^−1^ while chronoamperometry was carried out at −0.30 V for 20 h in 0.5 M H_2_SO_4_. Interestingly, MoS_1.99_Cu_0.01_ displayed high electrocatalytic activity with an increase in the reaction time. Similarly, after 1000 cycles of cycling stability analysis, we observed a further 0.140 V reduction in the onset potential. Subsequently, this leads to enhancement in the current density from 1 mA cm^−2^ to 5.8 mA cm^−2^ after 20 h of static durability measurements. This indicates that no obvious anti-leaching or de-activation of intrinsic active sites was detected with a change in time. But in turn, the activity was found to increase gradually with the reaction time, which could occur preferably due to the activation of copper and S-edge sites, which triggers new active centers in MoS_2_ for the HER to occur.

## Conclusions

In conclusion, we successfully synthesized zerovalent copper substituted MoS_2−*x*_ nanostructures *via* a simple, straightforward, scalable colloidal synthesis approach. Different characterization techniques support the substitution of copper atoms into the sulphur sites. The elemental composition of the Cu doped MoS_2_ by XPS shows the presence of Mo, S, and Cu, and the concentration of Cu in the Cu doped MoS_2_ increases linearly with an increase in the amount of copper precursor. Furthermore, TEM analysis and EDS mapping support the presence of a uniform dispersion of atomic copper atoms, and a consistent gap was observed on the layers, which was due to the substitution of the copper atoms. The experimental observation was validated with the first-principles calculations to understand S-substituted Cu doping feasibility and estimate the formation energies, which supports Cu substitution at S sites. With increasing Cu amount in the product, Cu transitions from atomic to crystalline. Raman analysis shows a shift in the A_1g_ peaks, which can be attributed to increased Cu substitution, causing a negative strain in the MoS_2_ lattice. More importantly, the substitution of copper atoms into MoS_2−*x*_ reduced the overpotential of the electrocatalyst significantly. The HER durability studies confirmed the triggering of new active sites, which activated the catalyst gradually with progress in the reaction time. This new finding offers a simple method to readily derive metal substituted 2D materials for catalytic applications with potential possibilities in several other areas such as electronics, energy storage, and functional device fabrications.

## Experimental section

### Materials

Molybdenum hexacarbonyl, sulfur powder, tetrakis(acetonitrile)copper(i) hexafluorophosphate (97%), and 1-octadecene (ODE, technical grade 90%) were all purchased from Sigma-Aldrich. Oleylamine (OLA, >50%) was purchased from TCI. The solvents such as hexanes (certified ACS grade) were acquired from Fisher Scientific. Ethanol (200 proof) was brought from Decon Laboratories, Inc. All chemicals were used as received without further purification.

### Synthesis of S deficient colloidal MoS_2−*x*_ nanostructures

MoS_2−*x*_ nanostructures were synthesized using a hot injection colloidal method.^42^ Initially, S-ODE solution was prepared by mixing 3 mmol of sulphur powder and 20 mL of ODE in a round bottom flask under a continuous argon (Ar) flow for 30 minutes at room temperature. The temperature was further increased to 100 °C and the mixture was stirred for 30 minutes until a clear faint yellow solution was noticed. In another flask Mo-OLA solution was prepared by mixing 2 mmol of Mo(CO)_6_ and 30 mL of OLA under a continuous Ar flow. The mixture was initially purged at room temperature for 30 minutes, then heated to 250 °C at a rate of 7 °C min^−1^ and maintained until a brown colored solution was obtained. The synthesized S-ODE solution was then gradually added to the Mo-OLA solution using a syringe at 1 mL min^−1^. Then the temperature of the mixture was increased to 280 °C and the mixture was stirred continuously for 1 h. After completion of the reaction, the mixture was cooled rapidly by removing the reaction vessel from the heating mantel and left undisturbed under an Ar atmosphere. We observed fine black particles at the bottom of the reaction vessel.

### Doping of MoS_2−*x*_ nanostructures with Cu

The synthesized MoS_2−*x*_ nanoparticles were purged under Ar, heated to 100 °C, and maintained for 30 minutes. About 0.02 to 2 mmol of the copper precursor was then rapidly added to the synthesized MoS_2−*x*_ colloidal dispersion and stirred continuously for 1 hour at 100 °C. The obtained black powder precipitate was cooled under an argon atmosphere and quenched in hexane. The formed powders were further washed with an excess of a mixture of hexane and ethanol, centrifuged, and annealed at 450 °C under 15 vol% H_2_ in Ar for 30 min to decompose the residual surfactants completely.

### Characterization

Raman analysis was carried out by blending the synthesized powder with potassium bromide (KBr) using a mortar and pestle and compressed to form a pellet. The Raman and photoluminescence (PL) measurements were performed under 532 nm laser irradiation with 10–50% laser power. The derived samples' surface morphology was observed under a scanning electron microscope (SEM, FEI Quanta 400 ESEM FEG) with 10 eV electron voltage. Additionally, energy-dispersive X-ray (EDX) spectroscopy was carried out using an FEI SEM to estimate the synthesized material's elemental composition. X-ray photoelectron spectroscopy (XPS, PHI Quantera) was used to study the elements' bonding state and atomic proportion. Binding energy calibration was based on C 1s at 284.6 eV. A Rigaku smart lab X-ray diffractometer with Cu Kα radiation was used to analyze the X-ray diffraction pattern and the synthesized samples' crystal structure. HRTEM was carried using a TEAM 1 microscope located at NCEM at an LBNL facility operated at 80 kV under low dose conditions approximately 2 e^−^ per pixel per s. The TEAM 1 microscope has a nominal resolution of about 50 pm, and it is equipped with a K2 camera. EDS was carried using a JEOL 2100 operated at 200 kV. In both cases, the samples were prepared using nickel (Ni) grids, 300 mesh.

### Electrochemical measurements

Electrochemical measurements, including linear sweep voltammetry, Tafel analysis, and impedance spectroscopy, were carried out using a three-electrode system with a Pt wire and a Hg/Hg_2_SO_4_ as the counter and reference electrodes, respectively. The working electrodes were prepared by coating derived samples on glassy carbon electrodes followed by drying in a vacuum desiccator for 14 h. All samples were sonicated for 20 minutes before coating on the polished electrodes. To convert the Hg/Hg_2_SO_4_ electrode potential (*E*_Hg/Hg2SO4_) to the reversible hydrogen electrode (*E*_RHE_), the following eqn (3) was used:

3 *E*_RHE_ = *E*_Hg/Hg2SO4_ + 0.682 V + 0.059pH

We acquired impedance spectra (Nyquist plot) by sweeping the frequency from 1 MHz to 10 mHz at an AC amplitude of 10 mV using a three-electrode system. Tafel analysis was executed at a scan rate of 1 mV s^−1^.

### Computational methods

Density functional theoretical calculations were carried out using the Vienna *Ab initio* Simulation Package (VASP).^43,44^ The interaction between the ionic cores and electrons was described with the Projected Augmented Wave (PAW) method,^45^ and the exchange–correlation energy between the electrons was determined by the local density approximation parameterized by the Perdew–Burke–Ernzerhof functional.^46^ The Kohn–Sham wavefunctions were represented using a plane wave basis set with an energy cut off at 400 eV, and the structures were relaxed until the Hellman–Feynman forces on the atoms were less than 0.01 eV Å^−1^. The energy convergence threshold for the self-consistent step was set to 10^−4^ eV. A vacuum of 30 Å was included in a direction perpendicular to the sheet to minimize the interaction between periodic images.

To study the concentration-dependent Cu doped MoS_2_ structures (MoS_2−*x*_Cu_*x*_), we constructed a 3 × 3 × 1 for *x* = 1/9, and 2 × 3 × 1 for *x* = 1/2 supercells. The Brillouin zone integrations in the primitive were sampled by 12 × 12 × 1 Monkhorst pack of *k*-points whereas those for the 3 × 3 × 1 and 2 × 3 × 1 supercells were sampled with 3 × 3 × 1 and 4 × 3 × 1 *k*-points, respectively. The *k*-point mesh sizes were converged within 5 meV per atom variation in total energy. The vibrational frequencies of the primitive and doped-supercell at the Brillouin zone center were estimated using the finite difference method as implemented in the VASP package. The structure was relaxed such that forces on the atoms were less than 0.001 eV Å^−1^ with an energy convergence threshold of 10^−7^ eV to achieve converged phonon frequencies.

## Author contributions

AKM & ZW designed and performed the experiments. HK helped in the analysis. JS and AKM carried out electrochemical measurements. TS and SS performed the DFT studies. FCRH, and HCB assisted with TEAM observations. RV, BIK, AKM, and PMA advised and supervised the project. All authors contributed to the writing and revisions of the article.

## Conflicts of interest

There are no conflicts to declare.

## Supplementary Material

NA-003-D0NA01064B-s001
