# Supplementary material for: Substitution of copper atoms into defect-rich molybdenum sulfides and their electrocatalytic activity
Source: Nanoscale Adv. 2021 Jan 26;3(6):1747–57. doi: 10.1039/d0na01064b (PMC9419841; doi:10.1039/d0na01064b)
Supplement: NA-003-D0NA01064B-s001 [file NA-003-D0NA01064B-s001.pdf]

Electronic Supplementary Material (ESI) for Nanoscale Advances.
This journal is © The Royal Society of Chemistry 2021

Supplementary Information

Substitution of Copper Atoms into Defect-Rich Molybdenum Sulfide and their Electrocatalytic Activity

Zixing Wang,[a] *Harikishan Kannan,*[a] *Tonghui Su,*[a] *Jayashree Swaminathan,*[a] *Sharmila N. Shirodkar,*[a] *Francisco C Robles Hernandez,*[a,b] *Hector Calderon Benavides,*[c] *Robert Vajtai,*[a,d] *Boris I. Yakobson,*[a] *Ashokkumar Meiyazhagan,**[a] *Pulickel M. Ajayan**[a]

[a] Department of Materials Science and NanoEngineering, Rice University, Houston, TX 77005, USA.

Email: ma37@rice.edu; ajayan@rice.edu

[b] Department of Mechanical Engineering Technology, University of Houston, Houston, Texas 77204-4020, United States

[c] Departamento de Física, ESFM-IPN, Ed. 9, Instituto Politécnico Nacional UPALM, Mexico D.F., Mexico 07738

[d] Interdisciplinary Excellence Centre, Department of Applied and Environmental Chemistry, University of Szeged, Rerrich Béla tér 1, Szeged, H-6720, Hungary

XPS Studies of MoS_{2-x} synthesized using wet chemical approach

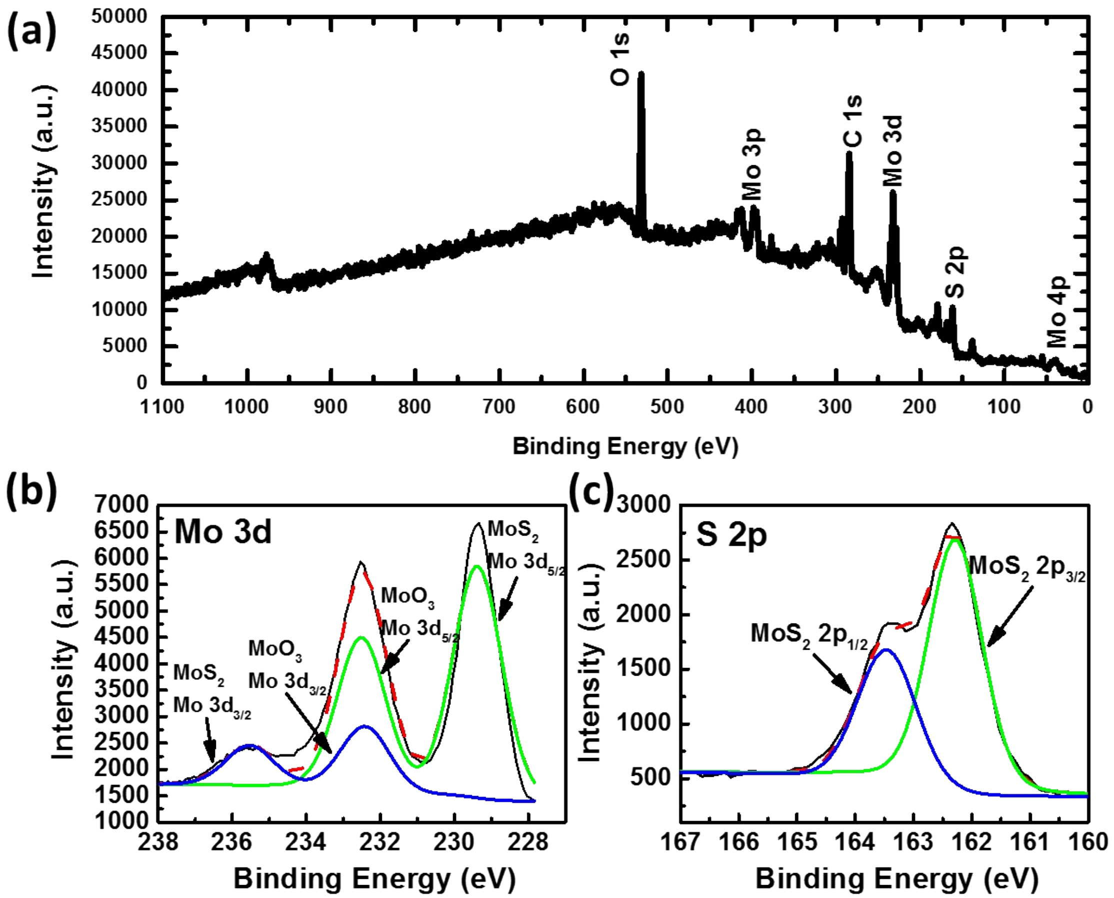

Figure S1 XPS spectra of colloidal synthesized MoS_{2-x} nanoparticles. (a) XPS survey spectra. The product contains 31.2 at% Mo, 37.5 at% S, and 31.2 at% O. (b) Mo 3d spectra of colloidal synthesized MoS_2 nanoparticles. Mo $3d_{5/2}$ peak for MoS_2 and MoO_3 are located at 229.4 eV and 232.4 eV respectively. 78.1 at% of Mo comes from MoS_2 and 21.9 at% Mo comes from MoO_3. (c) S 2p spectra of colloidal synthesized MoS_2 nanoparticles. The S intensity comes only from MoS_2, where $2p_{3/2}$ peak is located at 162.3 eV and $2p_{1/2}$ peak is located at 163.5 eV.

XPS Studies of $MoS_{1.99}Cu_{0.01}$ synthesized using wet chemical approach

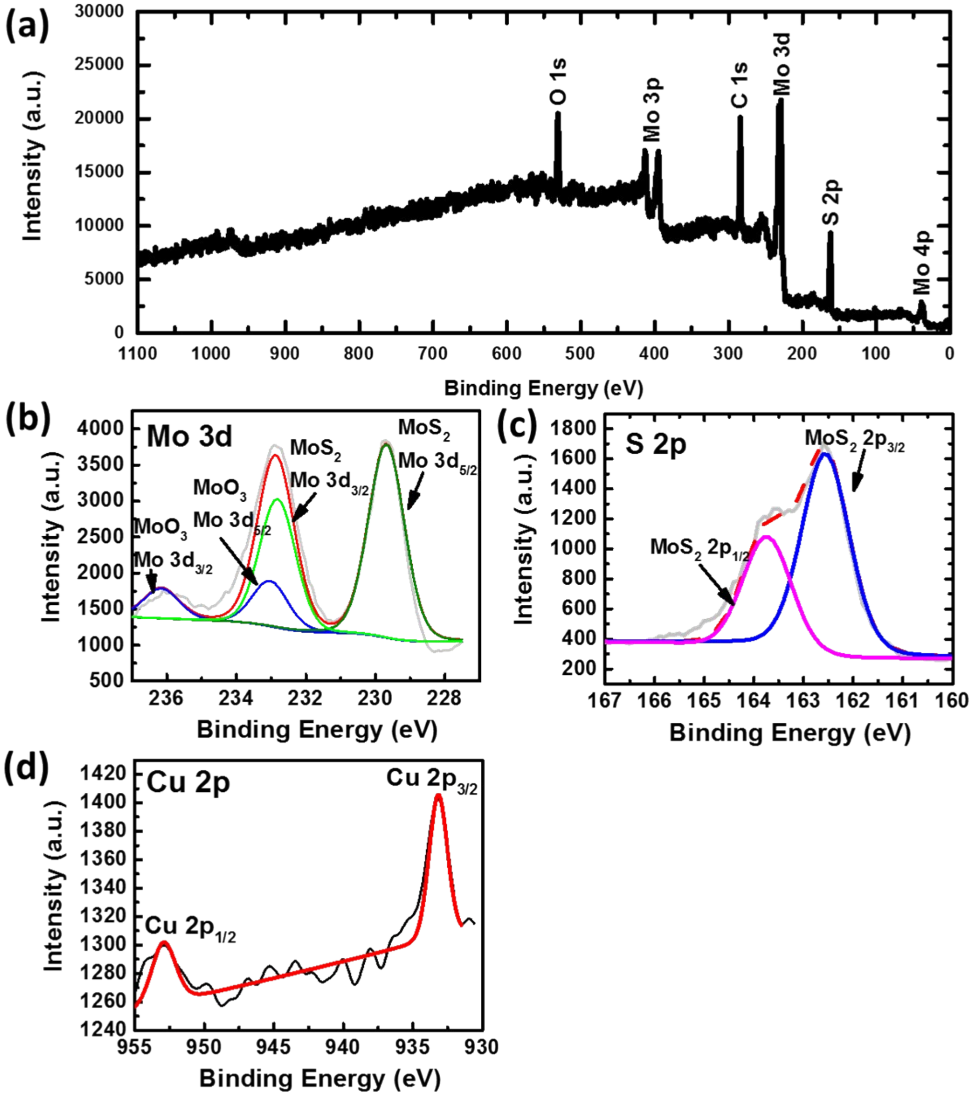

Figure S2 XPS spectra of $MoS_{1.99}Cu_{0.01}$ with tetrakis(acetonitrile)copper (I) hexafluorophosphate decomposed at 100 ^{o}C. (a) XPS survey spectra. The product contains 23.4 at% Mo, 33.7 at% S, 0.6 at% Cu and 42.3 at% O. (b) Mo 3d spectra of. Mo $3d_{5/2}$ peak for MoS_2 and MoO_3 are located at 229.7 eV and 233.0 eV respectively. 61 at% of Mo comes from MoS_2 and 39 at% Mo comes from MoO_3. (c) S 2p spectra of $MoS_{1.99}Cu_{0.01}$. The S intensity comes only from MoS_2, where $2p_{3/2}$ peak is located at 162.6 eV. (d) Cu 2p spectra of $MoS_{1.99}Cu_{0.01}$. The Cu signals come exclusively from Cu(0) or Cu(I). Since the Cu(0) and Cu(I) XPS peaks are close in position (< 0.1 eV difference in position), it is difficult to distinguish the two materials. The Cu $2p_{3/2}$ peak is located at 933.3 eV.

XPS Studies of $MoS_{1.9}Cu_{0.1}$ synthesized using wet chemical approach

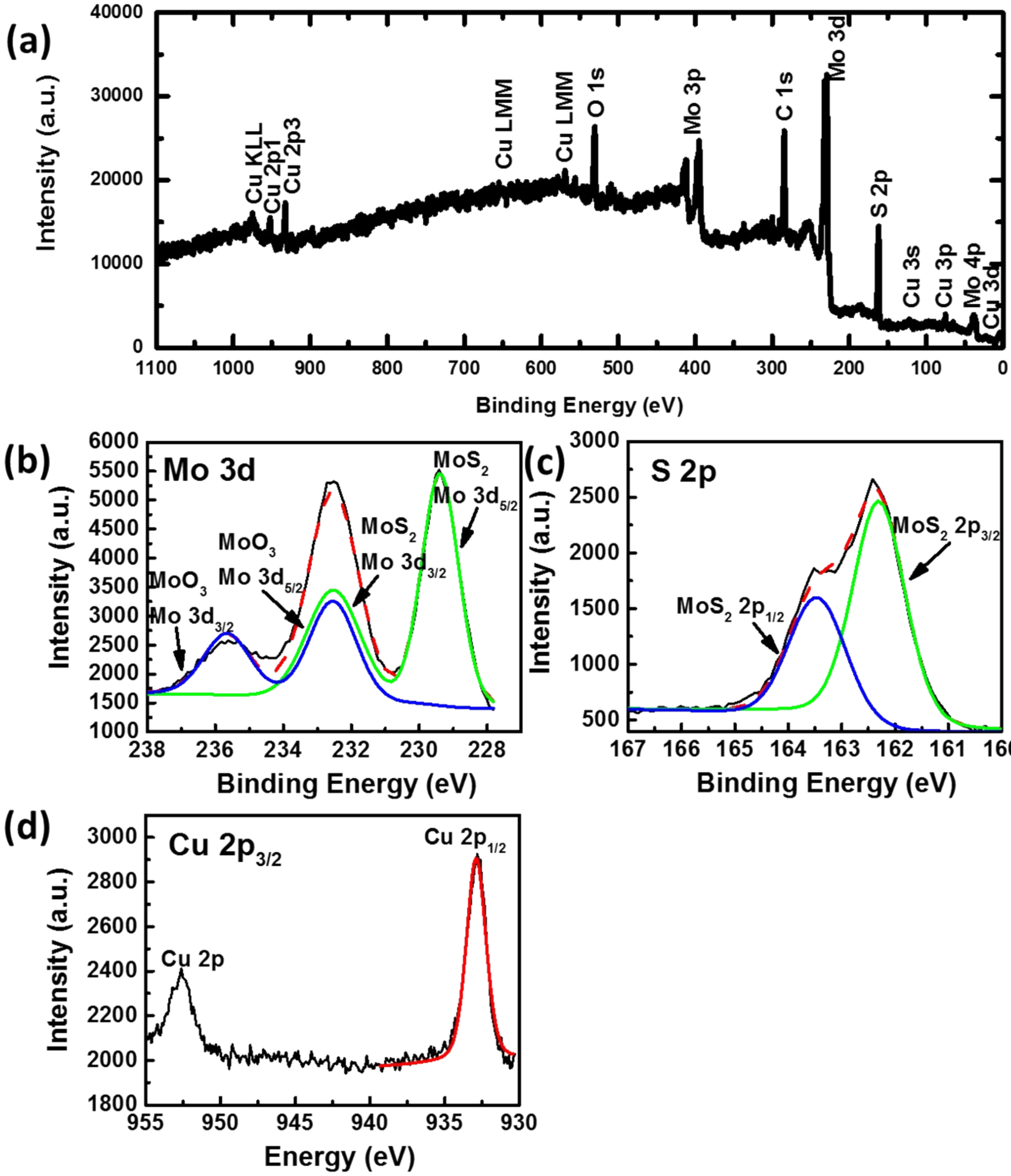

Figure S3 XPS spectra of $MoS_{1.9}Cu_{0.1}$ with tetrakis(acetonitrile)copper (I) hexafluorophosphate decomposed at 100 °C. (a) XPS survey spectra. The product contains 24.6 at% Mo, 35.6 at% S, 2.8 at% Cu and 37.0 at% O. (b) Mo 3d spectra of $MoS_{1.9}Cu_{0.1}$. Mo $3d_{5/2}$ peak for MoS_2 and MoO_3 are located at 229.4 eV and 232.4 eV respectively. 82 at% of Mo comes from MoS_2 and 18 at% Mo comes from MoO_3. (c) S 2p spectra of $MoS_{1.9}Cu_{0.1}$. The S intensity comes only from MoS_2, where $2p_{3/2}$ peak is located at 162.3 eV. (d) Cu 2p spectra of $MoS_{1.9}Cu_{0.1}$. The Cu $2p_{3/2}$ peak for Cu(0) or Cu(I) is located at 932.9 eV.

XPS Studies of MoS_1Cu_1 synthesized using wet chemical approach

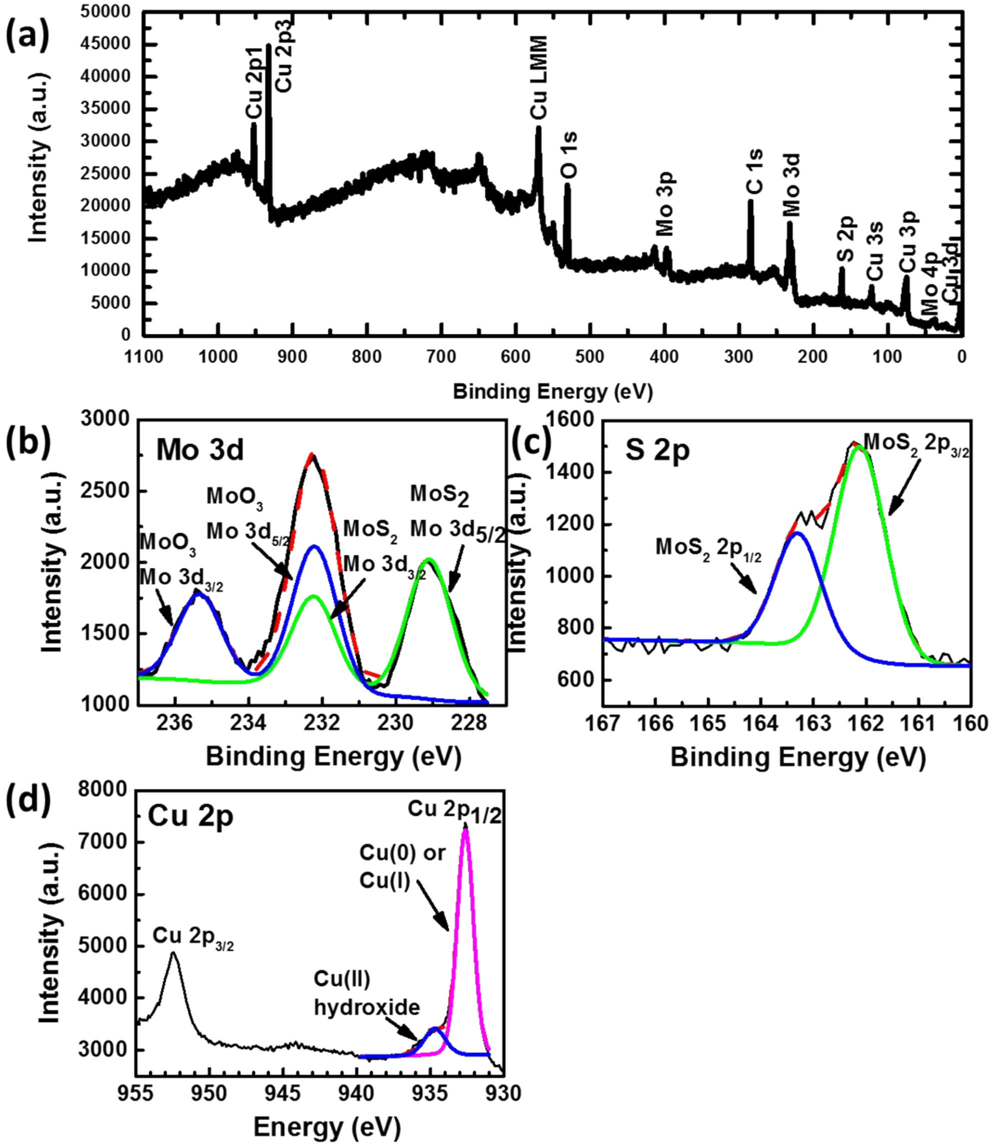

Figure S4 XPS spectra of colloidal synthesized MoS_1Cu_1 with tetrakis(acetonitrile)copper (I) hexafluorophosphate decomposed at 100 °C. (a) XPS survey spectra. The product contains 11.0 at% Mo, 15.7 at% S, 19.8 at% Cu and 53.5 at% O. (b) Mo 3d spectra. Mo $3d_{5/2}$ peak for MoS_2 and MoO_3 are located at 229.1 eV and 232.2 eV respectively. 51 at% of Mo comes from MoS_2 and 49 at% Mo comes from MoO_3. (c) S 2p spectra of MoSCu. The S intensity comes only from MoS_2, where $2p_{3/2}$ peak is located at 162.1 eV. (d) Cu 2p spectra of MoSCu. The Cu signals come from Cu(0) or Cu(I), and Cu(II) hydroxide. The Cu $2p_{3/2}$ peak for Cu(0) or Cu(I) is located at 932.6 eV

and for Cu(II) hydroxide is located at 934.7 eV. The Cu contains 86 at% Cu(0) or Cu(I) and 14 at% Cu(II) hydroxide.

XPS Studies of MoS_1Cu_1 synthesized using wet chemical approach

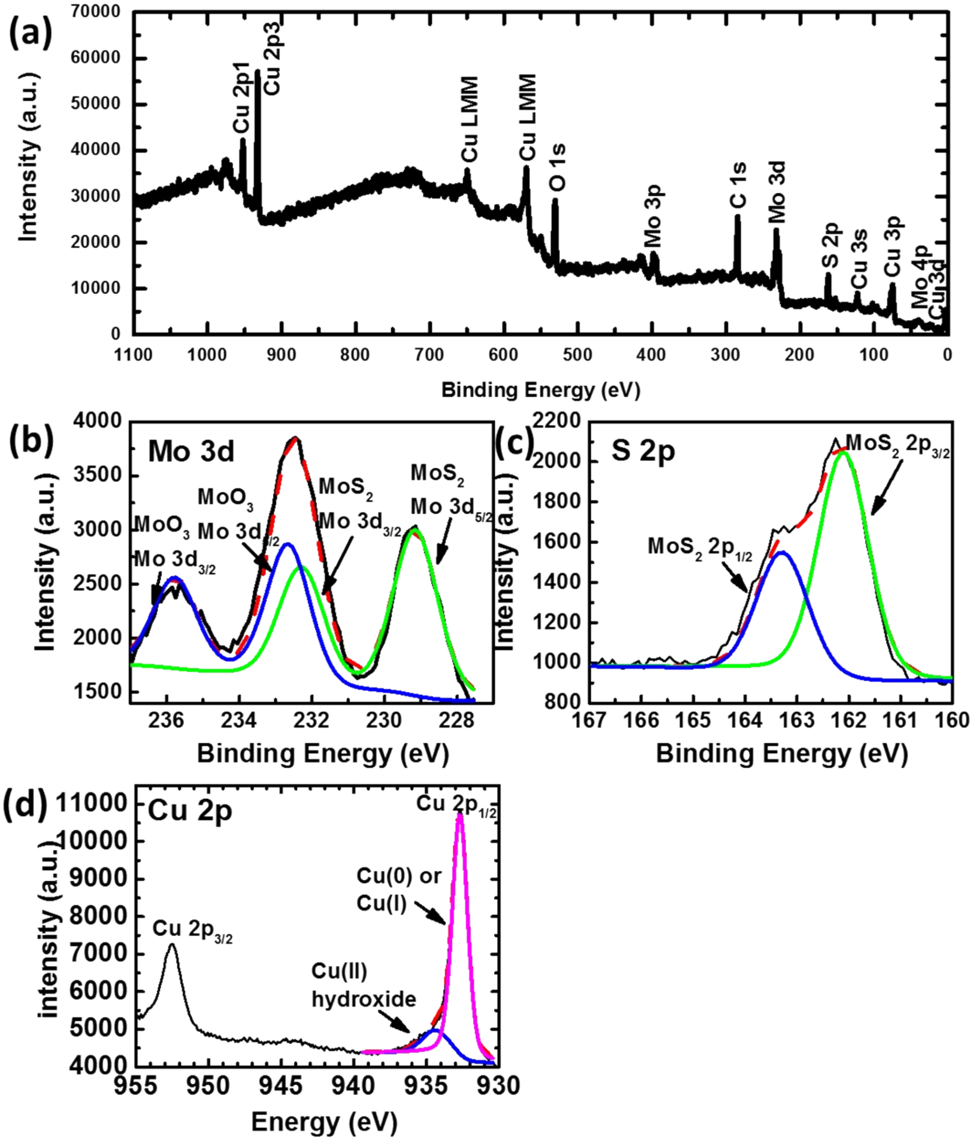

Figure S5 XPS spectra of colloidal synthesized MoS_1Cu_1 with tetrakis(acetonitrile)copper (I) hexafluorophosphate decomposed at 200 ^oC. (a) XPS survey spectra. The product contains 12.1 at% Mo, 16.3 at% S, 18.6 at% Cu and 53.0 at% O. (b) Mo 3d spectra of MoS_1Cu_1. Mo $3d_{5/2}$ peak for MoS_2 and MoO_3 are located at 229.2 eV and 232.6 eV respectively. 56 at% of Mo comes from MoS_2 and 44 at% Mo comes from MoO_3. (c) S2p spectra of MoS_1Cu_1. The S intensity is due to

MoS_2, where $2p_{3/2}$ peak is located at 162.2 eV. (d) Cu 2p spectra. The Cu signals display Cu(0) or Cu(I), and Cu(II) hydroxide. The Cu $2p_{3/2}$ peak for Cu(0) or Cu(I) is located at 933.3 eV and Cu(II) hydroxide is located at 935.5 eV. The Cu contains 76 at% Cu(0) or Cu(I) and 24 at% Cu(II) hydroxide.

TEM observation of the $MoS_{1.99}$-$Cu_{0.01}$

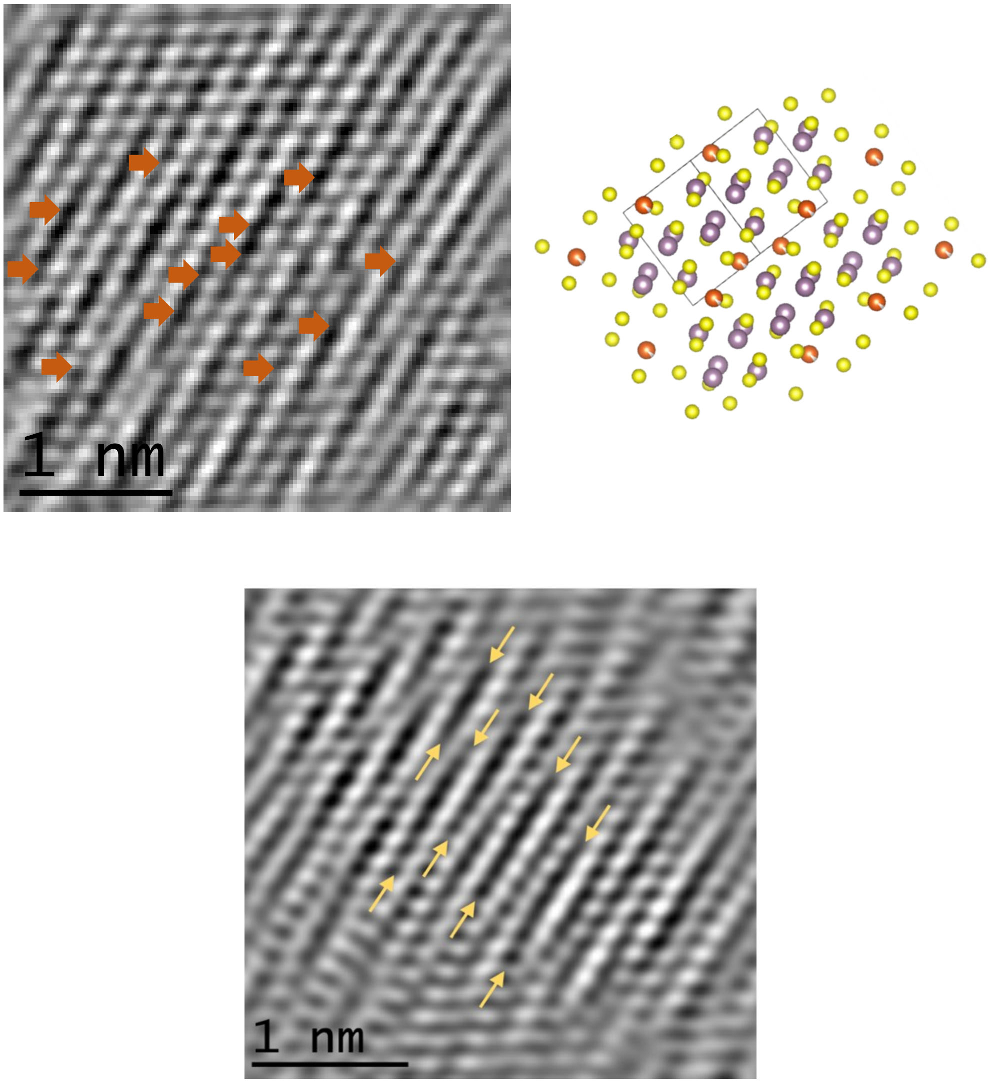

Figure S6 TEM observation of the $MoS_{1.99}$-$Cu_{0.01}$. Scheme: S (yellow), Mo (purple), Cu (orange) showing spaces in between the layers. The observed gaps are due to the presence of copper atoms.

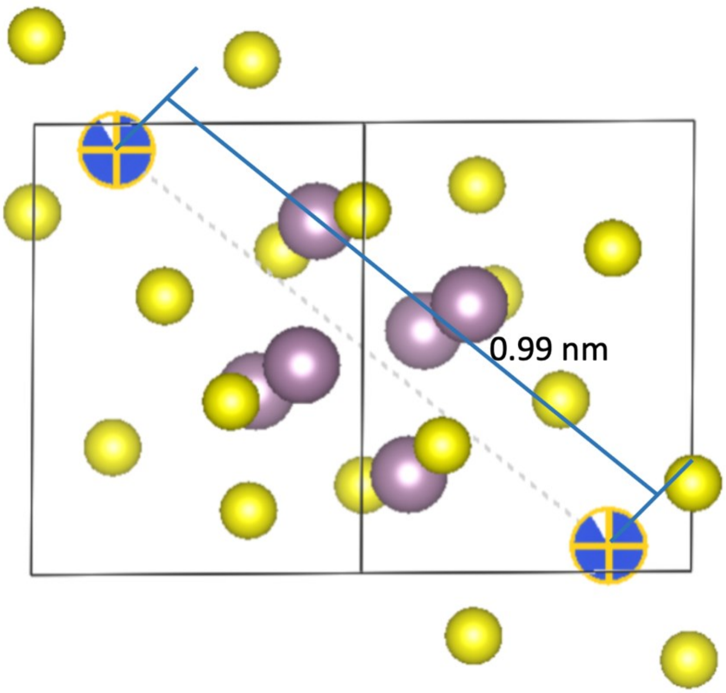

Figure S7 The measured distance between the Cu atoms is approximately 0.994 nm.

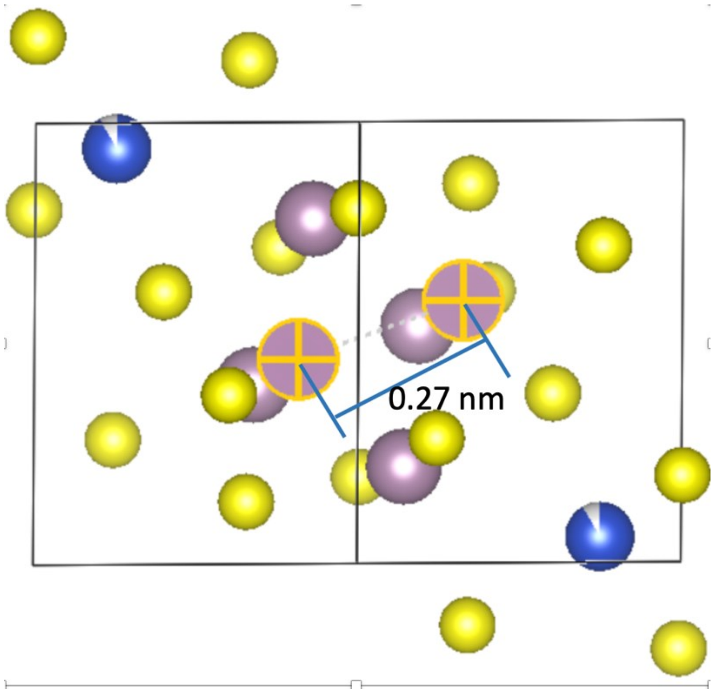

Figure S8 The measured Mo-S-Mo atoms is approximately 0.27 nm.

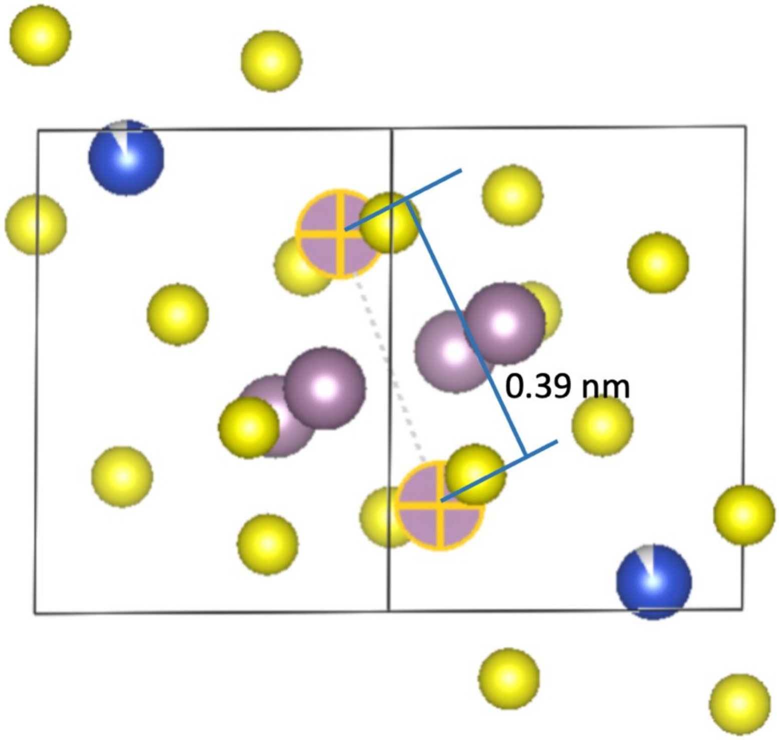

Figure S9 The measured S-Mo-S atoms is approximately 0.39 nm.

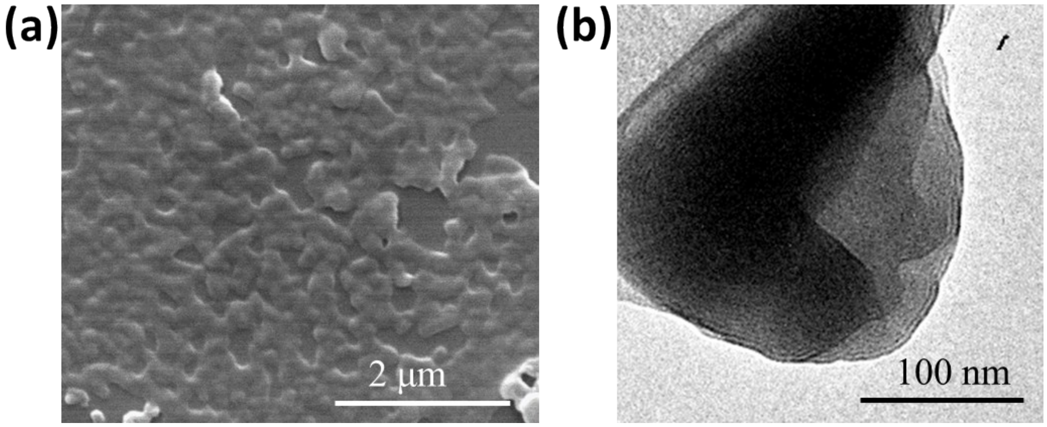

Figure S10 (a) SEM image of the $MoS_{1.99}Cu_{0.01}$ on Si wafer. A continuous thin layer of sheets can be observed. (b) TEM image of $MoS_{1.99}Cu_{0.01}$ displaying layered crystalline structure at the edges of the cluster.

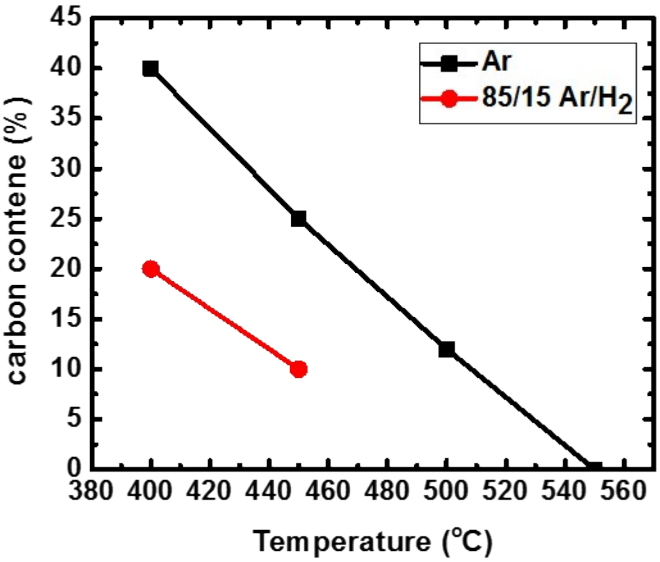

Figure S11 carbon content in the Cu doped MoS_2 samples when annealed in either Ar or 85/15 vol/vol Ar/H_2 atmosphere at temperatures ranging from 400 °C to 550 °C for 30 mins. The carbon content is determined by EDS. Ar/H_2 is more effective in removing the carbon from the sample. However, caution should be taken since the H_2 will reduce MoS_2 nanosheets and produce defects in the material. All carbon content can be removed at 550 °C under Ar atmosphere.

Effect of temperature and increase in proportion of copper precursor in formation of $MoS_{1.99}Cu_{0.01}$

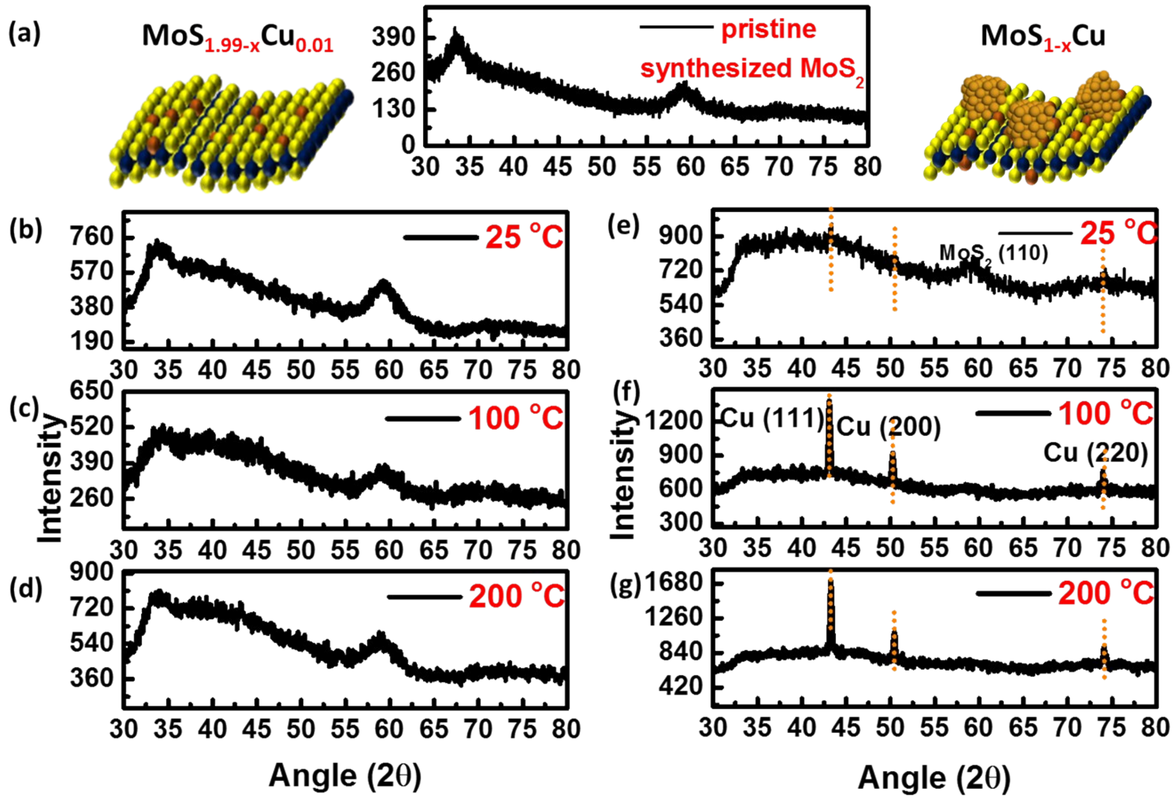

Figure S12 XRD spectra of (a) colloidal synthesized MoS_2. Left inset: cartoon of expected structure of $MoS_{1.99}Cu_{0.01}$. Right inset: cartoon of expected structure of $MoS_{1.99}Cu$. (b – d) $MoS_{1.99}Cu_{0.01}$ with tetrakis(acetonitrile)copper (I) hexafluorophosphate decomposed at (b) 25 ^{o}C, (c) 100 ^{o}C, and (d) 200 ^{o}C. The faint peak observed at 43.25^{o} corresponds to the prominent peak of Cu crystal. However, due to low Cu concentration, the Cu XRD peaks are faint. (e – g) $MoS_{1-x}Cu$ with tetrakis(acetonitrile)copper (I) hexafluorophosphate decomposed at (e) 25 ^{o}C, (f) 100 ^{o}C, and (g) 200 ^{o}C.

Distinct peaks of Cu crystal observed from samples synthesized at 100 ^{o}C, and 200 ^{o}C imply an optimal tetrakis(acetonitrile)copper (I) hexafluorophosphate decomposition temperature between 100-200 ^{o}C. The sharp peaks of Cu (marked by orange dashed lines, PDF # 00-026-1116) indicate the Cu crystals in the sample are highly crystalline, making the MoS_2 nanostructures faint in comparison.

Reaction pathway for synthesis of Cu doped MoS_x

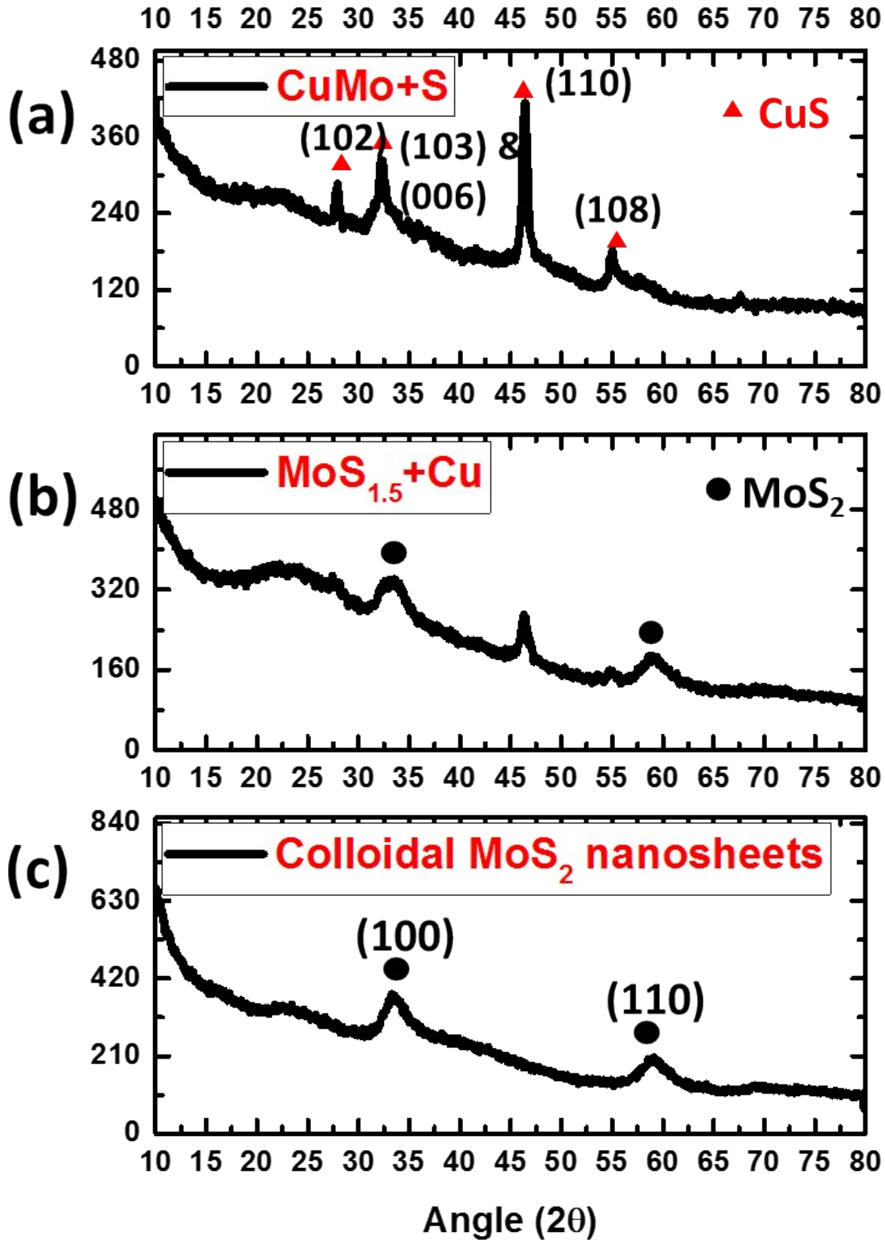

Figure S13 XRD spectra of Cu, Mo, S synthesized under different conditions at 300 °C. (a) tetrakis(acetonitrile)copper (I) hexafluorophosphate was added to Mo-OAm at 1:1 molar ratio at 150 °C and reacted for 30 minutes. Then S-ODE was added to Cu, Mo-OAm solution and reacted at 300 °C. Distinct CuS peaks are observed from the spectra. This indicates a kinetically favored reaction between Cu and S to form CuS than MoS_2. Thus, it is necessary to create a stable MoS_2 structure before Cu can be added to the reaction. (b) A 1 mole of tetrakis(acetonitrile)copper (I) hexafluorophosphate was added to synthesize 1 mol of $MoS_{1.5}$ at 300°C. Other than the spectra

from MoS_2 nanoparticles, peaks corresponding to CuS is also observed, signifying, the thermal activation energy is sufficient to form CuS compound at 300 °C. (c) XRD spectra corresponding to MoS_{2-x} nanosheets synthesized through colloidal synthesis.

Raman studies of $MoS_{1.99}Cu_{0.01}$ synthesized at different temperature

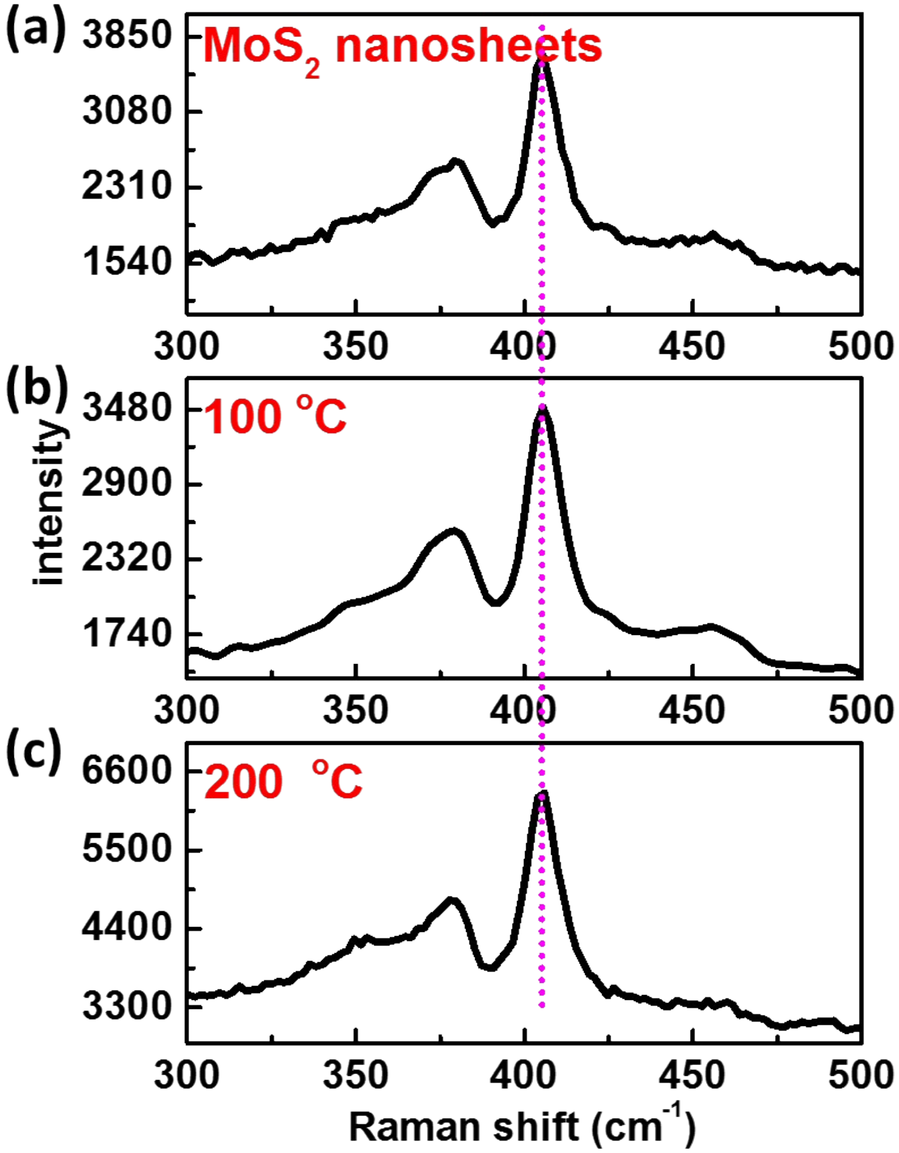

Figure S14 Raman spectra of (a) colloidal synthesized MoS_{2-x} and (b-c) $MoS_{1.99}Cu_{0.01}$ with tetrakis(acetonitrile)copper (I) hexafluorophosphate decomposed at (b) 100 °C, and (c) 200 °C. The A1g peak from (a), (b), and (c) are seen at 405.82, 405.80, and 405.74 cm^{-1} respectively.

Raman studies of $MoS_{1.9}Cu_{0.1}$ synthesized at different temperature

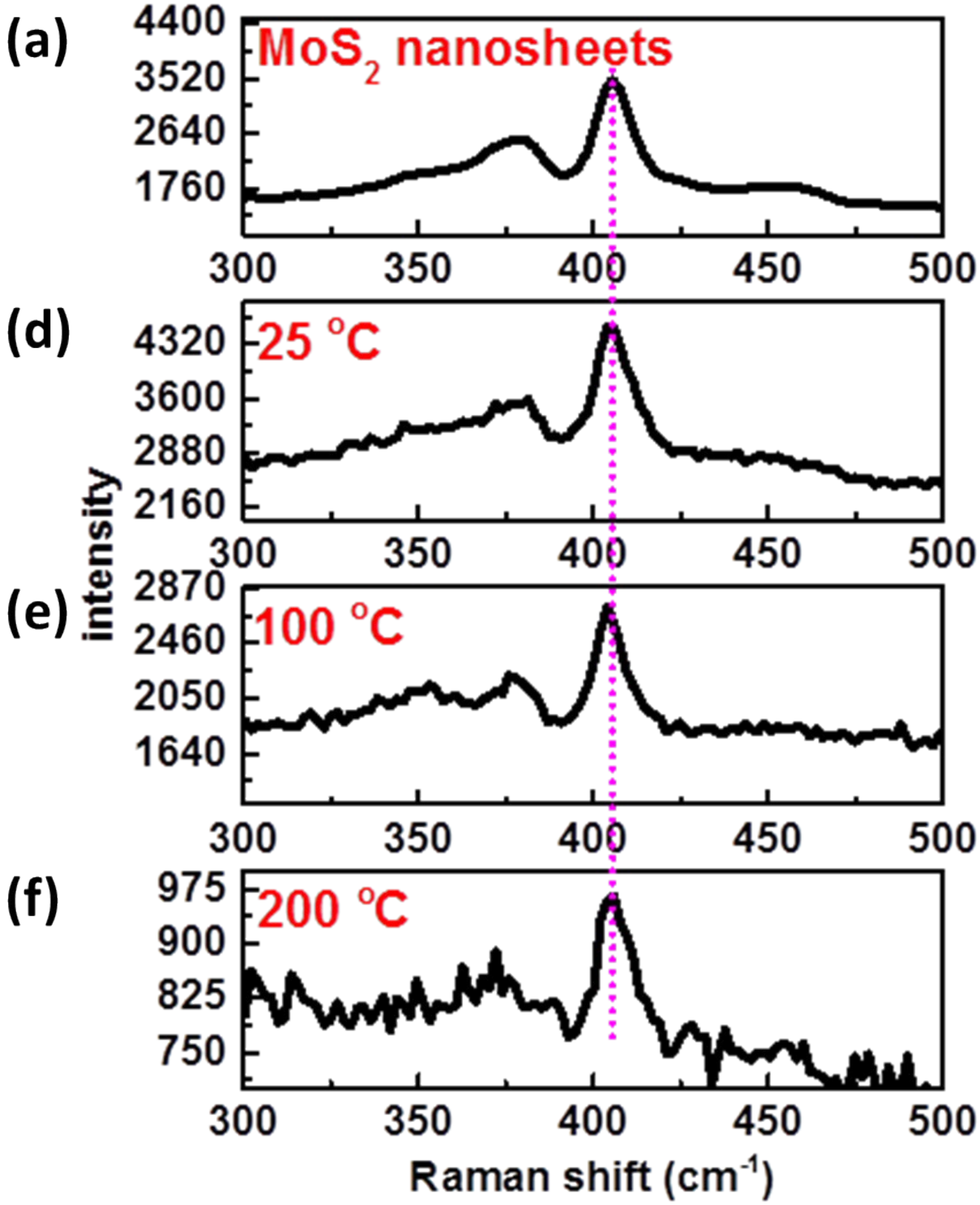

Figure S15 Raman spectra of (a) colloidal synthesized MoS_{2-x} and (b-d) $MoS_{1.9}Cu_{0.1}$ with tetrakis(acetonitrile)copper (I) hexafluorophosphate decomposed at (b) 25 ^{o}C, (c) 100 ^{o}C, and (d) 200 ^{o}C.

Raman studies of MoS_1Cu_1 synthesized at different temperature

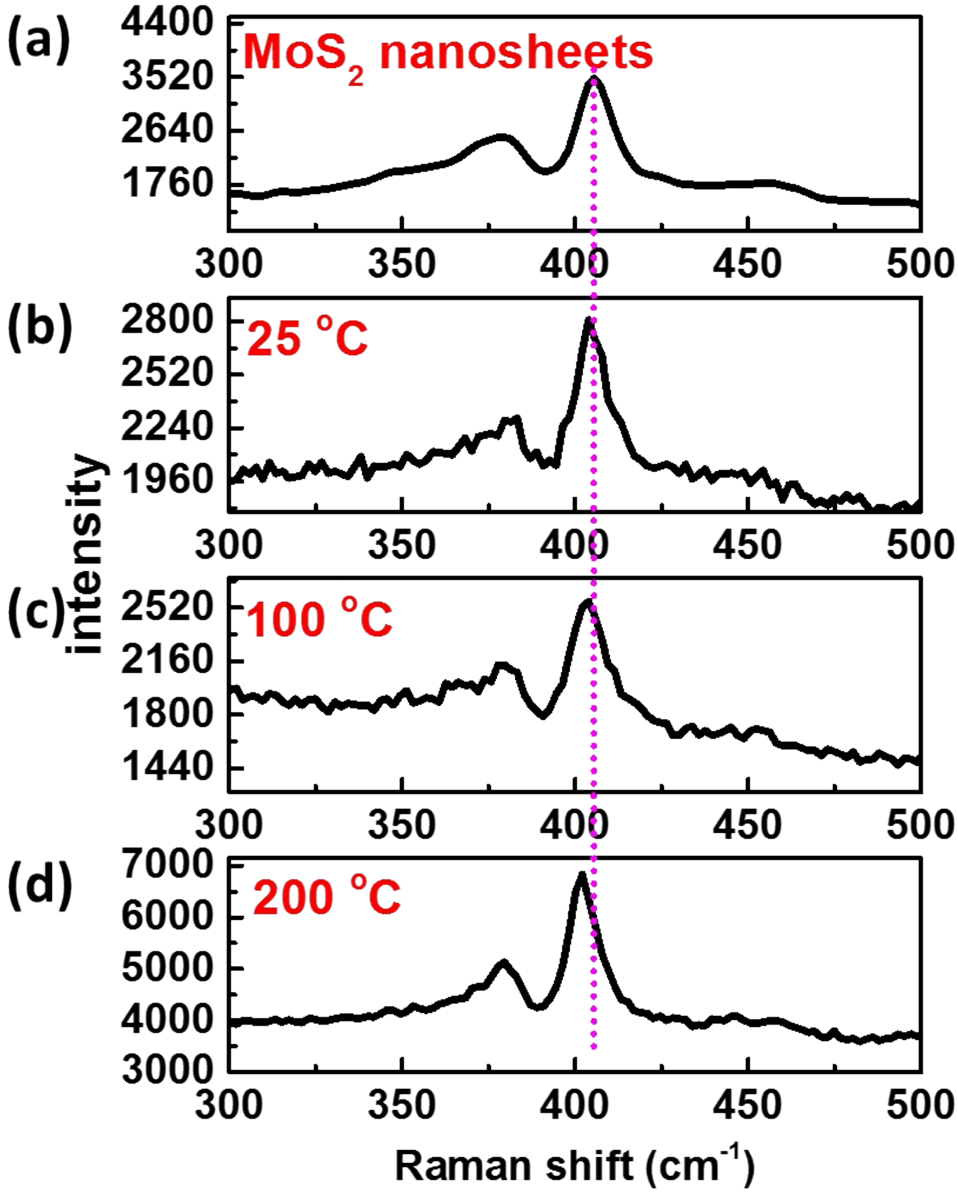

Figure S16 Raman spectra of colloidal synthesized (a) MoS_{2-x} nanostructures, (b-d) MoS_1Cu_1 with tetrakis(acetonitrile)copper (I) hexafluorophosphate decomposed at (b) 25 °C, (c) 100 °C, and (d) 200 °C. A1g peak position for synthesized MoS_2 nanostructures is 405.80 cm^{-1}. The peak position for tetrakis(acetonitrile)copper (I) hexafluorophosphate decomposed at 25 °C is 404.86 cm^{-1}, at 100 °C is 403.90 cm^{-1}, and at 200 °C is 402.40 cm^{-1}. A total shift of 3.4 cm^{-1} occurred upon Cu addition at 200 °C.

SEM EDAX observation of synthesized nanoparticles

EDAX observation of MoS_{2-x} nanostructures

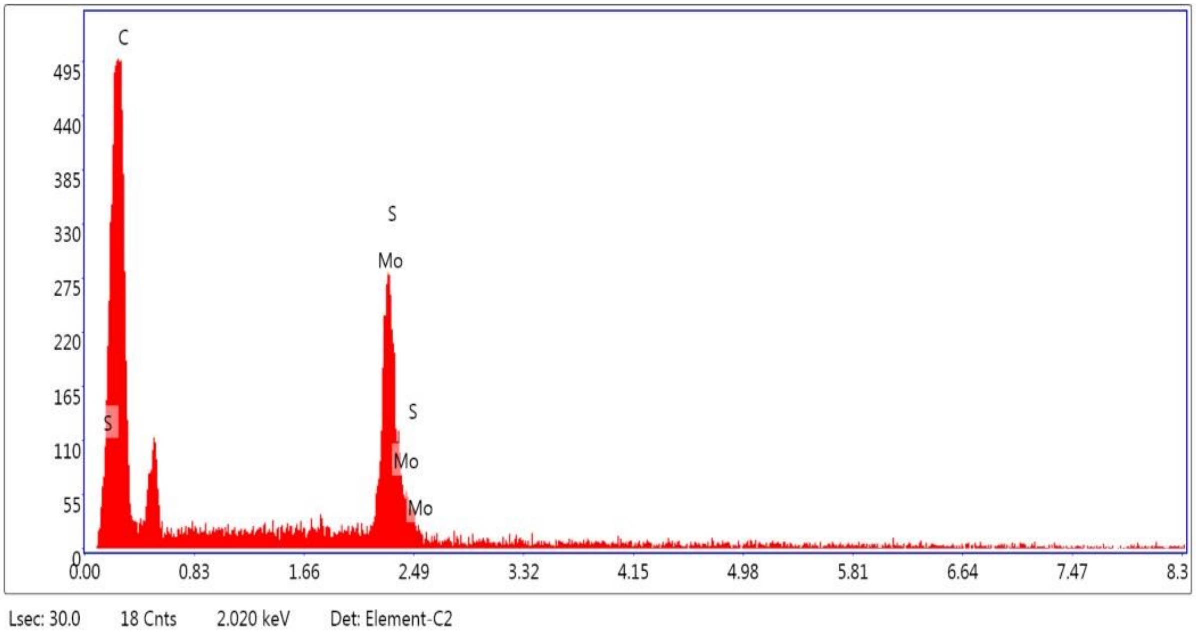

Figure S17 EDAX analysis of MoS_{2-x} sample shows presence of Molybdenum and sulfur

Formation of Cu doped MoS at 100°C

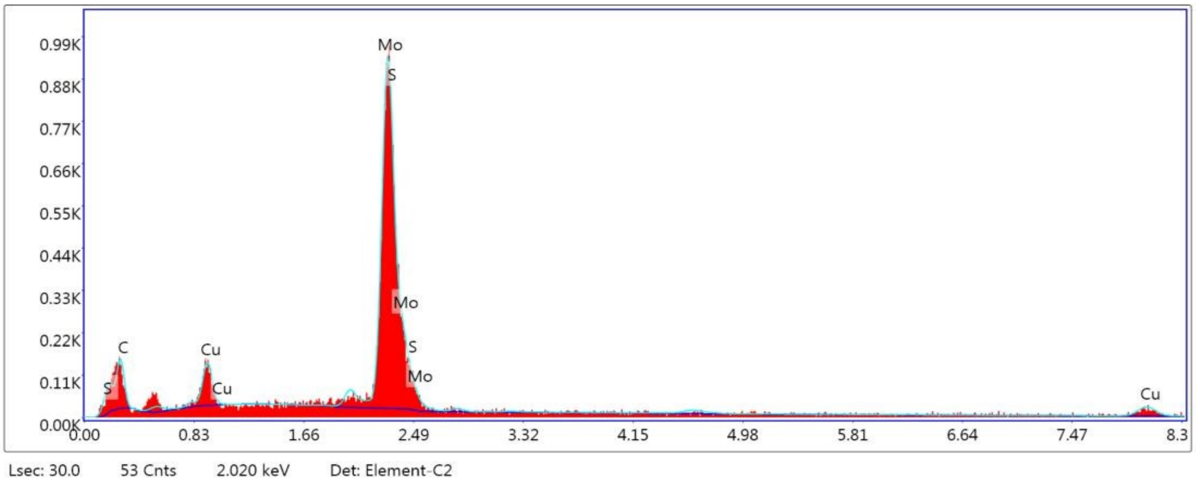

Figure S18 EDAX analysis of Cu doped MoS_{2-x} synthesized at 100°C shows presence of Molybdenum, copper and sulfur

Formation of CuS at 300°C

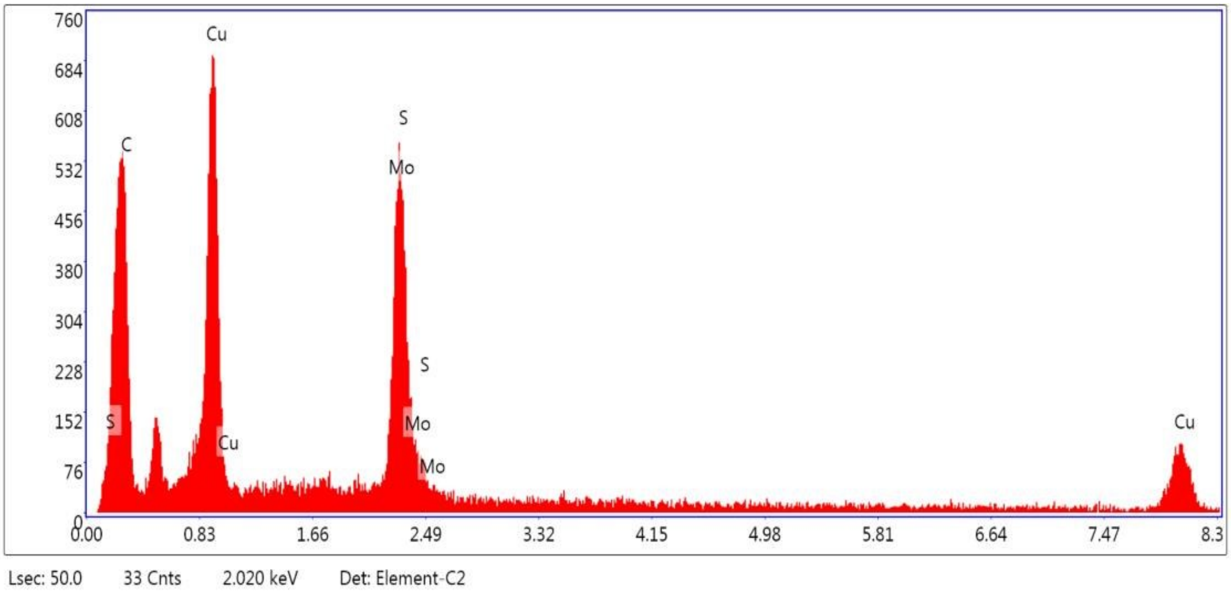

Figure S19 EDAX analysis of Cu doped MoS_{2-x} synthesized at 300°C displays presence of Molybdenum, along with higher percentages of sulfur and copper.

PL studies of $MoS_{1.99}Cu_{0.01}$ synthesized at different temperature

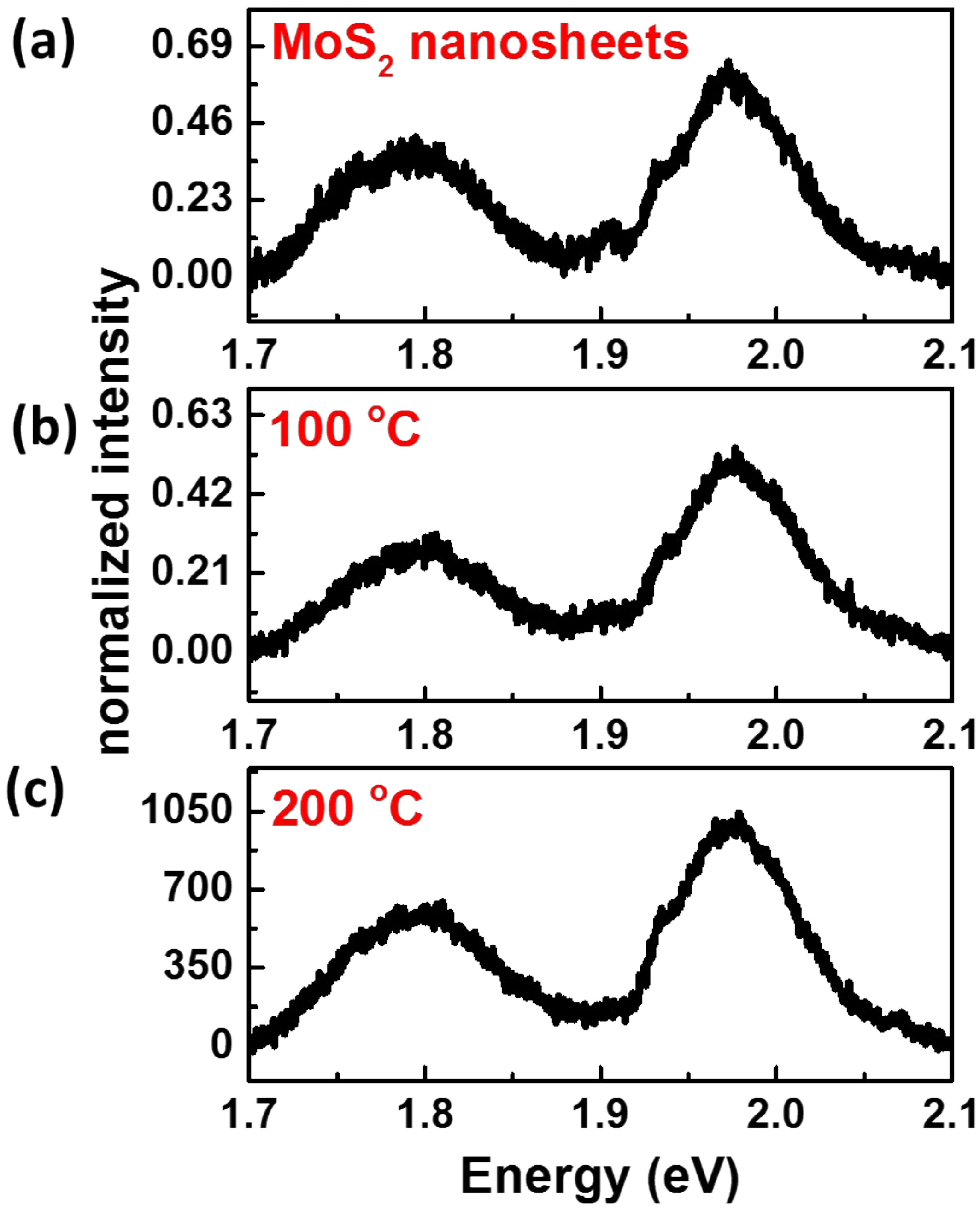

Figure S20 PL spectra of (a) colloidal synthesized MoS_{2-x} and (b-c) $MoS_{1.99}Cu_{0.01}$ with tetrakis(acetonitrile)copper (I) hexafluorophosphate decomposed at (b) 100 ^{o}C, and (c) 200 ^{o}C.

PL studies of $MoS_{1.9}Cu_{0.1}$ synthesized at different temperature

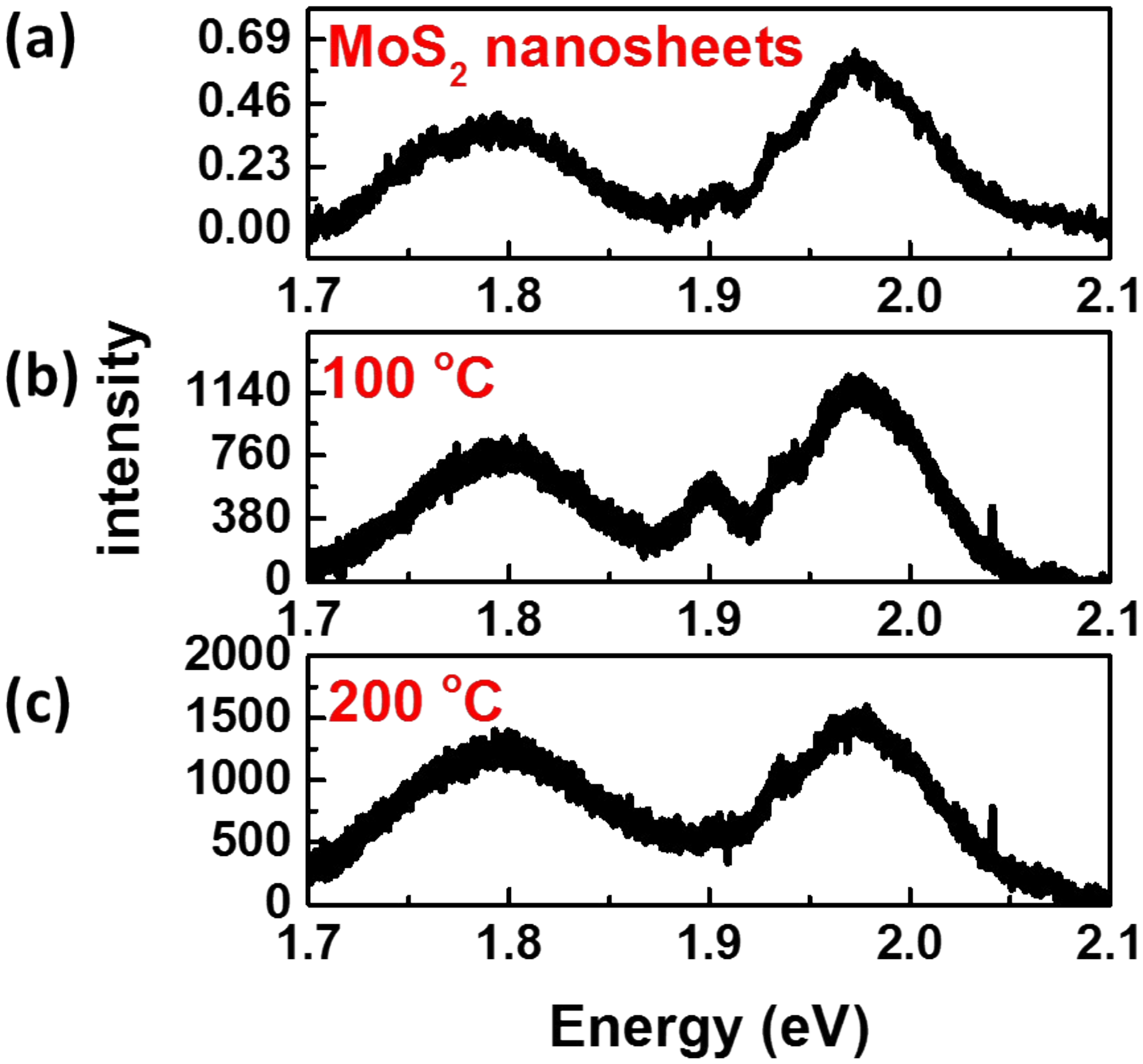

Figure S21 PL spectra of (a) colloidal synthesized MoS_{2-x} and (b-d) $MoS_{1.9}Cu_{0.1}$ with tetrakis(acetonitrile)copper (I) hexafluorophosphate decomposed at (b) 100 ^{o}C, and (c) 200 ^{o}C.

PL studies of MoS_1Cu_1 synthesized at different temperature

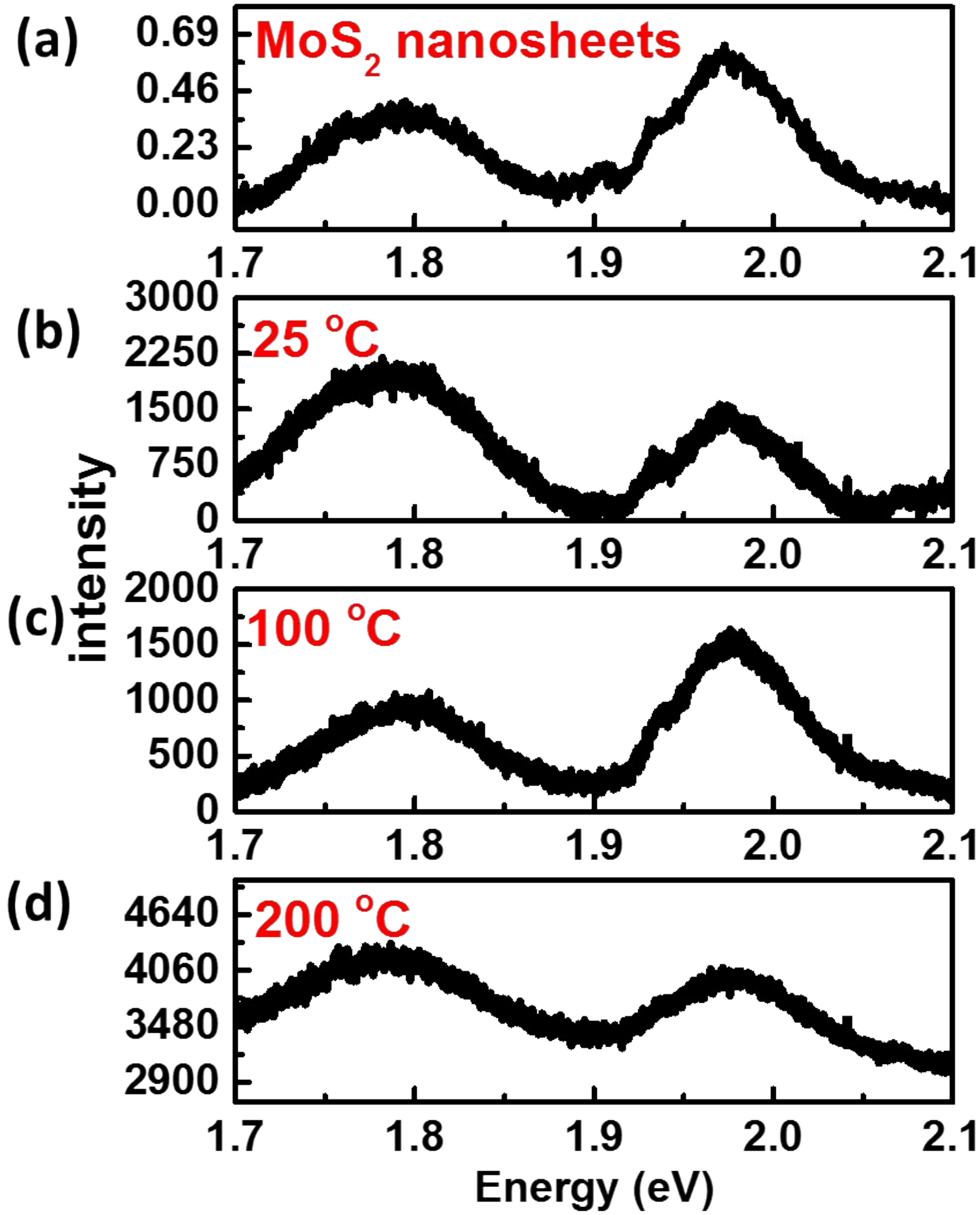

Figure S22 PL spectra of colloidal synthesized (a) MoS_{2-x} nanostructures, (b-d) MoS_1Cu_1 with tetrakis(acetonitrile)copper (I) hexafluorophosphate decomposed at (b) 25 °C (c) 100 °C, and (d) 200 °C.

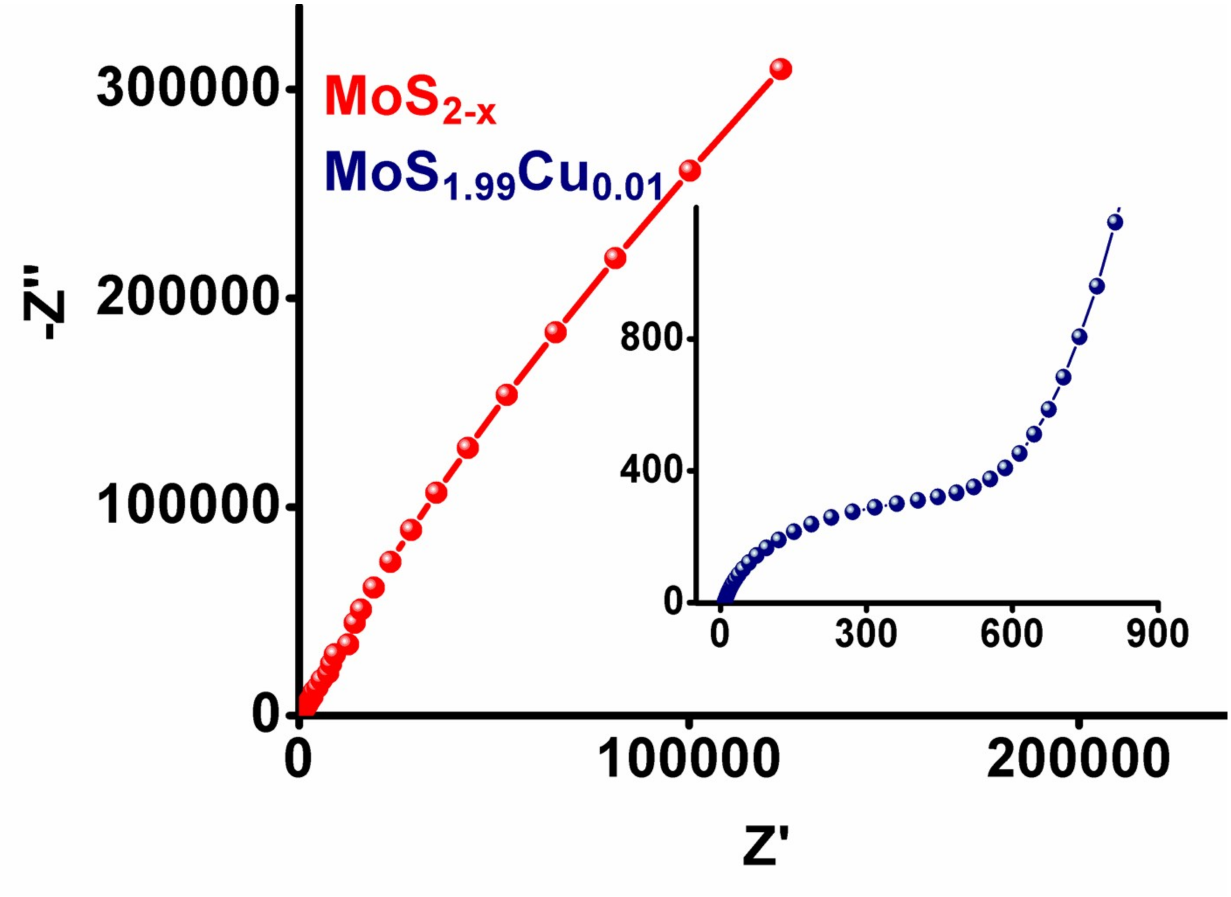

Figure S23. Nyquist plot of MoS_{2-x} and $MoS_{1.99}$ $Cu_{0.01}$

Table S1 Molar ratio of copper oxide and molybdenum oxide to $MoS_{2-x}Cu_x$ in the final product prepared under different reaction conditions.

**Sample**	**Cu Decomposition Temp (^oC)**	**CuO: MoO_3: $MoS_{2-x}Cu_x$ (molar ratio)**
**MoS_2 nanostructures**	NA	0: 0.44: 1
**$MoS_{1.99}Cu_{0.01}$**	100	0: 0.75: 1
**$MoS_{1.9}Cu_{0.1}$**	100	0: 0.30: 1
**MoS_1Cu_1**	100	0.07: 0.56: 1
**MoS_1Cu_1**	200	0.12: 0.57: 1

Table S2 Recent reports on catalytic performance of heteroatom-doped MoS_2-based catalysts

Dopant	Doping concentration (atomic %)	Onset potential [η, mV]	Tafel slope (mV. dec^{-1})	Reference
**Palladium**	1	-0.06	62	1
**Zinc**	4.33	-0.13	51	2
**Copper**	8.71	-0.16	68	2
**Nickel**	10.45	-0.19	89	2
**Iron**	10.77	-0.25	148	2
**Cobalt**	10.94	-0.24	141	3
**Nitrogen**	41	-0.3	77	4
**Copper**	**0.01**	**-0.26 (Initial cycle) -0.12 (After 1000 cycles)**	**75**	**Current work**

References

1) Zhaoyan Luo, Yixin Ouyang, Hao Zhang, Meiling Xiao, Junjie Ge, Zheng Jiang, Jinlan Wang, Daiming Tang, Xinzhong Cao, Changpeng Liu & Wei Xing, chemically activating MoS_2 via spontaneous atomic palladium interfacial doping towards efficient hydrogen evolution, Nat. Comm. 2018, 9, 2120 (1-8).
2) Yi Shi, Jiong Wang, Chen Wang, Ting-Ting Zhai, Wen-Jing Bao, Jing-Juan Xu, Xing-Hua Xia, and Hong-Yuan Chen, Hot Electron of Au Nanorods Activates the Electrocatalysis of Hydrogen Evolution on MoS_2 Nanosheets, J. Am. Chem. Soc. 2015, 137, 7365-7370.
3) Congrong Wang, Shun Wang, Jianguo Lv, Yuxuan Ma, Yongqi Wang, Gaoliang Zhou, Mingsheng Chen, Min Zhao, Xiaoshuang Chen, Lei Yang, Co doped MoS_2 as Bifunctional Electrocatalyst for Hydrogen Evolution and Oxygen Reduction Reactions, Int. J. Electrochem. Sci., 14 (2019) 9805-9814.
4) Qian Yang, Zegao Wang, Lichun Dong, Wenbin Zhao, Yan Jin, Liang Fang, Baoshan Hu, and Mingdong Dong, Activating MoS_2 with Super-High Nitrogen-Doping Concentration as, Efficient Catalyst for Hydrogen Evolution Reaction, J. Phys. Chem. C 2019, 123, 10917-10925
